# Atherosclerotic plaque fibroblasts derive from adventitial and medial *Pdgfra*-lineage-positive cells and predominantly maintain fibroblast identity

**DOI:** 10.1093/cvr/cvag183

**Published:** 2026-09-09

**Authors:** Guanliang Li, Sebastiaan E J Asselberghs, Baixue Yu, Cong Feng, Paul-Lennard Mendez, Dimitri Kasakovski, Pieter Goossens, Tia M Ackermans, Renée J H A Tillie, Marion J Gijbels, Lieve Temmerman, He Xu, Marjo M P C Donners, Dlzar A Kheder, Sidrah Maryam, Han Jin, Chenyi Xue, Sikander Hayat, Austin C Conklin, Casey E Romanoski, Mauro Giacca, Clint L Miller, Muredach P Reilly, Ming Chen, Rafael Kramann, Barend Mees, Andrew H Baker, Judith C Sluimer

**Affiliations:** Department of Pathology, Cardiovascular Research Institute Maastricht (CARIM), Maastricht University Medical Center (MUMC), P. Debyelaan 25, 6229 HX Maastricht, The Netherlands; Department of Pathology, Cardiovascular Research Institute Maastricht (CARIM), Maastricht University Medical Center (MUMC), P. Debyelaan 25, 6229 HX Maastricht, The Netherlands; Department of Vascular Surgery, CARIM, MUMC, Maastricht, The Netherlands; Department of Pathology, Cardiovascular Research Institute Maastricht (CARIM), Maastricht University Medical Center (MUMC), P. Debyelaan 25, 6229 HX Maastricht, The Netherlands; Department of Bioinformatics, College of Life Sciences, Zhejiang University, Hangzhou, China; Bioinformatics Center, The First Affiliated Hospital, Zhejiang University, Hangzhou, China; Department of Pathology, Cardiovascular Research Institute Maastricht (CARIM), Maastricht University Medical Center (MUMC), P. Debyelaan 25, 6229 HX Maastricht, The Netherlands; Department of Pathology, Cardiovascular Research Institute Maastricht (CARIM), Maastricht University Medical Center (MUMC), P. Debyelaan 25, 6229 HX Maastricht, The Netherlands; Department of Nephrology and Hypertension, Rheumatology and Immunology (Medical Clinic II), Medical Faculty RWTH Aachen, Pauwelsstraße 30, 52074 Aachen, Germany; Department of Pathology, Cardiovascular Research Institute Maastricht (CARIM), Maastricht University Medical Center (MUMC), P. Debyelaan 25, 6229 HX Maastricht, The Netherlands; Department of Pathology, Cardiovascular Research Institute Maastricht (CARIM), Maastricht University Medical Center (MUMC), P. Debyelaan 25, 6229 HX Maastricht, The Netherlands; Department of Pathology, Cardiovascular Research Institute Maastricht (CARIM), Maastricht University Medical Center (MUMC), P. Debyelaan 25, 6229 HX Maastricht, The Netherlands; Department of Pathology, Cardiovascular Research Institute Maastricht (CARIM), Maastricht University Medical Center (MUMC), P. Debyelaan 25, 6229 HX Maastricht, The Netherlands; Department of Medical Biochemistry, Amsterdam Cardiovascular Sciences: Atherosclerosis & Ischemic Syndrome, Amsterdam UMC Location University of Amsterdam, Amsterdam, the Netherlands; Amsterdam Infection and Immunity: Inflammatory Diseases, Amsterdam UMC Location University of Amsterdam, Amsterdam, the Netherlands; Department of Pathology, Cardiovascular Research Institute Maastricht (CARIM), Maastricht University Medical Center (MUMC), P. Debyelaan 25, 6229 HX Maastricht, The Netherlands; Department of Pathology, Cardiovascular Research Institute Maastricht (CARIM), Maastricht University Medical Center (MUMC), P. Debyelaan 25, 6229 HX Maastricht, The Netherlands; Department of Pathology, Cardiovascular Research Institute Maastricht (CARIM), Maastricht University Medical Center (MUMC), P. Debyelaan 25, 6229 HX Maastricht, The Netherlands; Department of Pathology, Cardiovascular Research Institute Maastricht (CARIM), Maastricht University Medical Center (MUMC), P. Debyelaan 25, 6229 HX Maastricht, The Netherlands; Department of Nephrology and Hypertension, Rheumatology and Immunology (Medical Clinic II), Medical Faculty RWTH Aachen, Pauwelsstraße 30, 52074 Aachen, Germany; Department of Pathology, Cardiovascular Research Institute Maastricht (CARIM), Maastricht University Medical Center (MUMC), P. Debyelaan 25, 6229 HX Maastricht, The Netherlands; Central Laboratory, Tianjin Medical University General Hospital, Tianjin, China; Division of Cardiology, Department of Medicine, Columbia University Irving Medical Center, Irving Institute for Clinical and Translational Research, Columbia University, New York, NY, USA; Department of Nephrology and Hypertension, Rheumatology and Immunology (Medical Clinic II), Medical Faculty RWTH Aachen, Pauwelsstraße 30, 52074 Aachen, Germany; Center of Excellence in Nephrology, University Hospitals Aachen, Cologne, Dusseldorf, Aachen, Germany; STEM Division, Northwest College, Powell, Wyoming, USA; The Department of Cellular and Molecular Medicine, The University of Arizona, Tucson, USA; Molecular Medicine, International Centre for Genetic Engineering and Biotechnology (ICGEB), Trieste, Italy; King's College London, British Heart Foundation (BHF) Centre of Research Excellence, London, UK; Department of Medicine, Surgery and Health Sciences, University of Trieste, Trieste, Italy; Department of Genome Sciences, University of Virginia, Charlottesville, Virginia, USA; Division of Cardiology, Department of Medicine, Columbia University Irving Medical Center, Irving Institute for Clinical and Translational Research, Columbia University, New York, NY, USA; Department of Bioinformatics, College of Life Sciences, Zhejiang University, Hangzhou, China; Bioinformatics Center, The First Affiliated Hospital, Zhejiang University, Hangzhou, China; Department of Nephrology and Hypertension, Rheumatology and Immunology (Medical Clinic II), Medical Faculty RWTH Aachen, Pauwelsstraße 30, 52074 Aachen, Germany; Center of Excellence in Nephrology, University Hospitals Aachen, Cologne, Dusseldorf, Aachen, Germany; Department of Internal Medicine, Nephrology and Transplantation, Erasmus Medical Center, Rotterdam, The Netherlands; Department of Vascular Surgery, CARIM, MUMC, Maastricht, The Netherlands; Department of Pathology, Cardiovascular Research Institute Maastricht (CARIM), Maastricht University Medical Center (MUMC), P. Debyelaan 25, 6229 HX Maastricht, The Netherlands; BHF Centre for Cardiovascular Science, Queen’s Medical Research Institute, University of Edinburgh, 47 Little France Crescent, Edinburgh EH16 4TJ, UK; Department of Pathology, Cardiovascular Research Institute Maastricht (CARIM), Maastricht University Medical Center (MUMC), P. Debyelaan 25, 6229 HX Maastricht, The Netherlands; Department of Nephrology and Hypertension, Rheumatology and Immunology (Medical Clinic II), Medical Faculty RWTH Aachen, Pauwelsstraße 30, 52074 Aachen, Germany; Center of Excellence in Nephrology, University Hospitals Aachen, Cologne, Dusseldorf, Aachen, Germany; BHF Centre for Cardiovascular Science, Queen’s Medical Research Institute, University of Edinburgh, 47 Little France Crescent, Edinburgh EH16 4TJ, UK

**Keywords:** Atherosclerosis, Fibroblasts, Single-cell RNA sequencing, Lineage tracing, Cell fate

## Abstract

**Aims:**

Fibroblasts are mesenchymal cells in the healthy vascular adventitia. In atherosclerosis, single-cell sequencing datasets suggest fibroblasts are abundant in plaques. However, their identity, origin, and fate during plaque progression remain unclear, which we aim to unravel here.

**Methods and results:**

To robustly define fibroblast identity, origin, and fate, we employed meta-analyses of 54 single-cell RNA sequencing libraries, including murine smooth muscle cell (*Myh11*) and endothelial cell (EC) (*Cdh5*) lineage reporter mice with and without atherosclerosis; human control and atherosclerotic arteries; and murine adventitia and atherosclerotic plaques processed separately from low-density lipoprotein (LDL) receptor knockout (*Ldlr^−/−^*) mice. These meta-analyses showed that murine and human plaque fibroblast identity was robustly defined by *Pdgfra*, *Pi16*, *Cygb*, and *Serpinf1* mRNA. Ninety-five percent of plaque fibroblasts do not derive from the *Myh11* lineage, while no *Cdh5*-lineage-positive cells were present in the fibroblast cluster. We identified five murine arterial fibroblast subsets in atherosclerotic murine aorta: progenitor fibroblasts, matrix fibroblasts, inflammatory fibroblasts, an EC-like fibroblast subset, detected in both adventitia and plaques, and *Col5a3^+^* fibroblasts, unique to the adventitia. We next studied fibroblast identity, origin, and fate using pseudotime analysis and *Pdgfra*-Cre^ERT2^/tdTomato lineage reporter mice (*Pdgfra* Lin^+^). Healthy *Pdgfra* Lin^+^ reporter mice showed predominant adventitial tdTomato expression, and infrequent medial and intimal *Pdgfra* Lin^+^ cells co-expressing MYH11 and PECAM1, respectively. The *Pdgfra* Lin^+^ plaque area increased with diet duration. *Pdgfra* Lin^+^ cells largely maintain fibroblast identity in the plaque, while <10% co-express SMC markers (MYH11, SM22α), or contribute to ACTA2^+^ cap cells. ECs gaining mesenchymal markers are transcriptionally distinct from *Cdh5*-lineage-negative fibroblasts gaining EC markers. Plaque-resident EC-like fibroblasts displayed a mesenchymal-to-endothelial transition transcriptome, which was induced in human primary fibroblasts *in vitro* by starvation, and dampened or reversed by IL1B, TGFB1, TGFB3, and oxidized LDL. Cross-species integration showed that all murine plaque fibroblasts were conserved in human atherosclerosis, with one additional subset partially resembling murine subsets, and three human-specific subsets. Importantly, human fibroblast subsets differentially correlated to human plaque traits, with EC-like fibroblasts correlating to plaque instability.

**Conclusion:**

Our results indicate that 95% of plaque-residing fibroblasts are *Myh11* Lin^−^ Plaque fibroblasts have a dual origin, predominantly adventitial *Pdgfra* Lin^+^ progenitor fibroblasts, with a minor contribution from medial *Pdgfra* Lin^+^  *Myh11^+^* SMCs. Most plaque fibroblasts maintain fibroblast identity. Murine plaque fibroblast subsets were conserved in human atherosclerosis. EC-like fibroblasts are linked to human plaque instability. Intervening in progenitor-to-specific fibroblast transitions could present a new avenue to promote plaque stability in atherosclerosis.

## Graphical Abstract

**Graphical Abstract:**
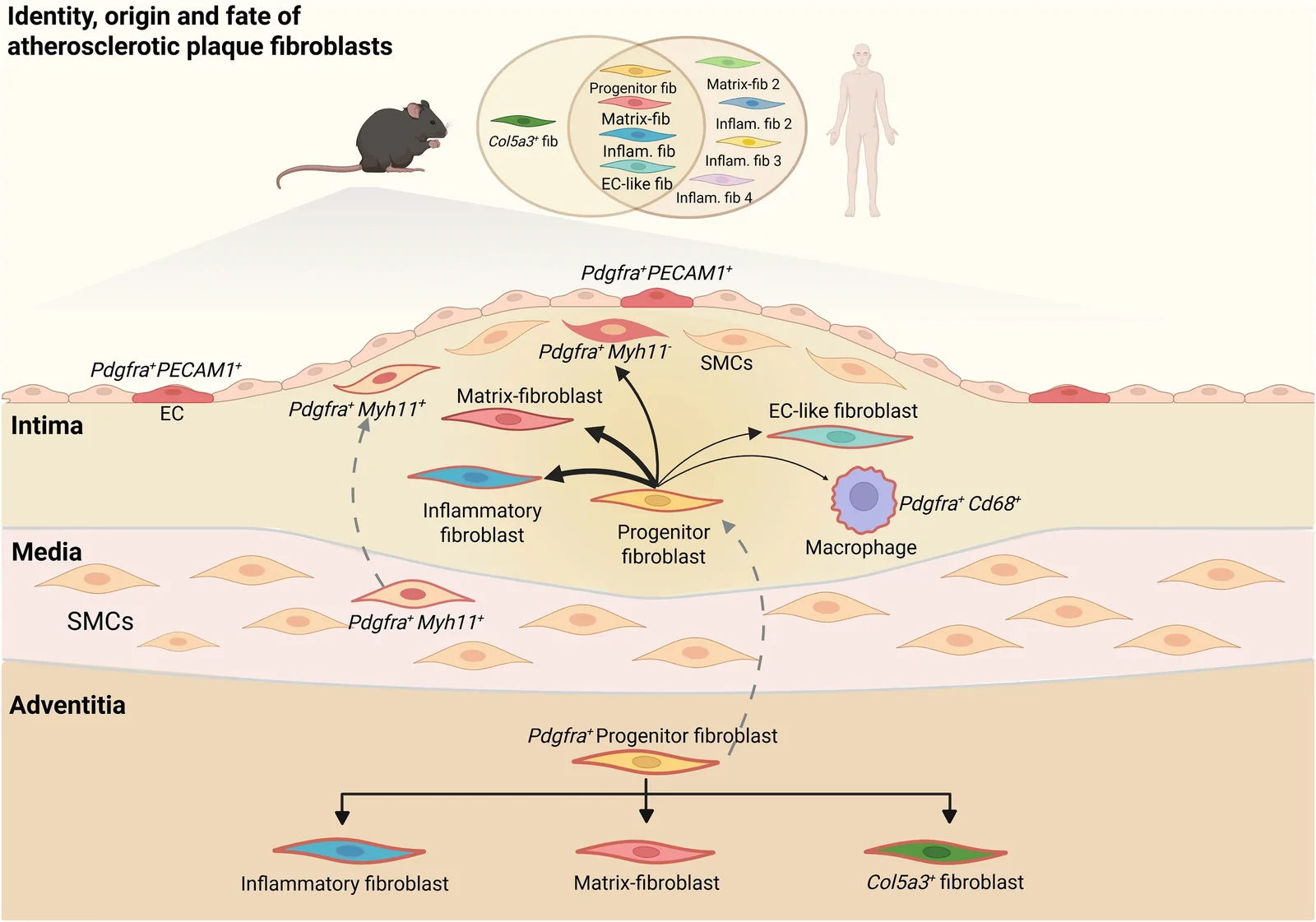
The figure was created with BioRender.com (Agreement number: BK299GJN66).

**Time of primary review: 4 days**

## 1. Introduction

By capturing the spectrum of phenotypes and functional states of diseased vascular cells *in vivo*, advances in single-cell RNA sequencing (scRNA-seq) have enabled the unravelling of the complexity of cell identity and heterogeneity in atherosclerosis. This has revealed increased phenotypic and functional heterogeneity among cells of the atherosclerotic plaque, such as for the mesenchymal smooth muscle cell (SMCs) populations.^1–5^ Interestingly, scRNA-seq studies also confirmed the abundant presence of another mesenchymal cell, the fibroblast, in the atherosclerotic plaque, previously only detected in the adventitia.^2,3,6,7^ These reports showed the presence of several fibroblast subsets in atherosclerotic plaques of the human and mouse aorta and coronary, carotid, and brachiocephalic arteries. It is important to note that the fibroblast-harbouring adventitial layer was often not removed in those studies before the vessels were digested for scRNA-seq, opening the possibility that parts of the identified fibroblast subsets could be of adventitial origin. Other studies have used protein expression of platelet-derived growth factor alpha (PDGFRA), fibroblast activation protein, fibroblast-specific protein 1, and vimentin^7,8^ to detect the intraplaque presence of fibroblast-like cells in intact tissue sections. However, none of these markers have been properly validated for the specificity of plaque fibroblasts, and many traditional fibroblast markers do not show a fibroblast-restricted mRNA expression pattern.^7^ In fact, our recent report shows that only PDGFRA and Dipeptidase 1 specifically marked adventitial fibroblasts, while traditional fibroblast markers appeared to mark SMCs even in healthy arteries.^7^ Adding to the ambiguity of historical data, the commonly used marker alpha-smooth muscle actin 2 (αSMA, or ACTA2) is not restricted to SMCs, as its name would suggest, but is also expressed by fibroblasts.^7^ Hence, several recent studies resort to using the term ‘fibroblast-like cells’,^9–11^ preventing unambiguous conclusions and comparison between studies. Thus, the identity and origin of plaque fibroblasts need to be accurately resolved.

Transcriptionally, we define plaque fibroblasts by high expression of established fibroblast markers, including Pdgfra and dipeptidase 1, with low expression of canonical SMC, endothelial, or immune-cell markers. Plaque fibroblasts could either differentiate from other cell types involved in atherosclerosis and/or migrate into the lesion from the adventitial layer. Previous studies suggested that SMCs or endothelial cells (ECs) are the origin of fibroblast-like cells in atherosclerotic plaque.^3,8^ As an indication of an SMC-related origin, two plaque cell types, myofibroblasts and fibromyocytes, express markers of both SMCs and fibroblasts.^9^ However, these differ in their proposed origin. Myofibroblasts are activated fibroblasts acquiring ACTA2 expression.^12^ For fibromyocytes, myosin heavy chain 11 (*Myh11*) lineage tracing confirmed their SMC origin.^3^ However, a (partial) fibroblast origin has not been studied with lineage tracing. Likewise, endothelial-to-mesenchymal transition (EndoMT) has been confirmed with endothelial lineage tracing using cadherin 5 (Cdh5),^8,13^ but whether these cells acquire a full or partial fibroblast identity is not known.

Considering the adventitial origin of plaque fibroblasts, the contribution of adventitial lymphocyte antigen 6a [LY6A, also known as stem cell antigen 1 (SCA-1)]-positive progenitor cells to plaque cells has been demonstrated; however, their definite contribution to fibroblasts specifically has not been proven.^14–18^ We previously reported that 96.5% of fibroblasts in healthy aortic adventitia express the stemness marker SCA-1, indicative of extensive fibroblast plasticity.^7,19^ However, SCA-1 expression is also observed in certain SMC, immune cell, and EC populations.^7,20–24^ As a result, studies into migration and differentiation of the general SCA-1^+^ progenitor cell population cannot be simply extrapolated to adventitial fibroblasts. A potential adventitial contribution to plaque fibroblasts has therefore not been clarified in atherosclerosis. Similarly, the fate of plaque fibroblasts is unclear. Fibroblasts are a known source to generate induced pluripotent stem cells.^25^ Given this plasticity, it is likely that fibroblasts have the capacity to differentiate into other cell types within the atherosclerotic plaque.

Our recent results identified PDGFRA as a robust marker of adventitial fibroblasts, and the availability of CRE-drivers of *Pdgfra*, *Myh11*, and *Cdh5* to induce inheritable fluorescent gene expression now allow to elucidate the origin of plaque fibroblasts using inducible genetic fate tracing. Thus, we employed *Pdgfra* lineage tracing, and scRNA-seq analysis of discrete murine adventitia and plaque tissue to explore the identity, origin, and fate of plaque fibroblasts. We substantiated this with scRNA-seq meta-analyses of murine *Myh11* and *Cdh5* lineage reporter mice, and of human control and atherosclerotic arteries.

## 2. Methods

### 2.1 Inclusion and ethics

All animal experiments were approved by the regulatory authorities at Maastricht University. Animal experiments were performed in compliance with Dutch governmental guidelines and European Parliament Directive 2010/63/EU on protection of animals used for scientific purposes. Human aorta and atherosclerotic plaque samples in carotid arteries for the AtheroMaas and the subcohort MaasHPS were collected as part of the Maastricht Pathology Tissue Collection.^26^ Storage, collection, and use of the tissue were in line with the Dutch Code for Proper Secondary Use of Human Tissue and the local Medical Ethical Committee (protocol number 16-4-181). This code (www.coreon.org/wp-content/uploads/2020/04/coreon-code-of-conduct-english.pdf) includes an opt-out arrangement and thus, tissues were not used in case of objection. Human studies conducted by Wirka *et al*.^3^ and Hu *et al*.^27^ were approved by the Stanford University Institutional Review Board and the Human Ethics Committee of Fuwai Hospital, Chinese Academy of Medical Sciences, respectively, and were conducted in accordance with the principles of the Declaration of Helsinki. Written informed consent was obtained from all participants before tissue collection.

### 2.2 Meta-analysis of scRNA-seq datasets from *Myh11* and *Cdh5* reporter mice

ScRNA-seq datasets were imported directly as filtered count matrices in accordance with the meta-analysis,^6^ and sample details of the original paper were described in Supplementary material online, Table  *S1*.^2,3,6,28,29^ Subsequent analysis was performed using R and the Seurat R package (v4.4.0).^30^ Data normalization was performed using the ‘NormalizeData’ method, followed by scaling using the ‘ScaleData’ method. Dimensionality reduction was performed using principal component analysis (PCA) on the 2000 most variable genes identified by the ‘FindVariableFeatures’ method. Harmony (v1.1.0)^31^ was used to integrate samples. Forty harmony embeddings were used for both graph-based clustering with the ‘FindCluster’ method (resolution = 0.4, selected using the Clustree R package^32^) and for uniform manifold approximation and projection (UMAP).

The ‘FindAllMarkers’ function was used to identify differentially expressed genes (DEGs) with the fold change and the Bonferroni-adjusted *P*-value for statistical measures. Identified clusters were categorized based on their marker gene expression as being SMC (*Cnn1*, *Myh11*, *Tagln*), fibromyocyte (*Vcam1*, *Tnfrsf11b*), Chondromyocyte (*Col2a1*, *Acan*), fibroblast (*Pdgfra*, *Pi16*, *Serpinf1*, *Cygb*), pericyte (*Rgs5*, *Notch3*, *Higd11b*), EC (*Cdh5*, *Pecam1*, *Vwf*), mesothelial cell (*Upk3b*, *Msln*, *Lrrn4*), Schwann cell (*Plp*, *Cnp*), T cell (*Cd3d*, *Cd3g*), B cell (*Igkc*, *Cd79b*, *Cd79a*), macrophage (*C1qa*, *C1qb*, *C1qc*), monocyte (*Itgam*, *Ccr2*, *Csf1r*), neutrophil (*S110a8*, *S110a9*, *Retnlg*), and dendritic cell (*Cd209a*, *Flt3*, *Ccr7*).

Lineage annotations in the included datasets were based on sorting the cells expressing the fluorescent reporter protein under control of the lineage promoters MYH11 for SMCs and CDH5 for ECs. *Myh11*-lineage-positive and *Myh11*-lineage-negative cells are abbreviated as *Myh11* Lin^+^ and *Myh11* Lin^−^, respectively, and *Cdh5*-lineage-positive and *Cdh5*-lineage-negative cells are abbreviated as *Cdh5* Lin^+^ and *Cdh5* Lin^−^, respectively. When referring to endogenous marker detection or RNA expression, we do not include ‘Lin’. Because the ZsGreen-based FACS sorting of lineage-traced cells by Pan *et al*.^2^ was subsequently reported to overestimate the *Myh11*-lineage-positive compartment, we incorporated the updated, algorithmically reassigned *Myh11* lineage annotation for the Pan *Ldlr^−/−^* dataset reported in their subsequent study.^33^ This correction was not available for the Pan *ApoE^−/−^* arm because a week 0 reference dataset was lacking. The updated *Myh11* lineage classification for the Pan *Ldlr^−/−^* dataset was then combined with the *Myh11* lineage classification from the Wirka *ApoE^−/−^* dataset to evaluate *Myh11* lineage proportions across cell types.

Subsequent fibroblast subset analyses were conservatively focused on FACS-sorted Myh11-lineage-negative (*Myh11* Lin^−^) fibroblasts. These cells were further reclustered using similar methods, with harmony parameters theta = 4 and max_iter = 20. Clusters were identified with a resolution of 0.15, which was selected using the Clustree package, and markers were detected using ‘FindAllMarkers’ and the COSG^34^ package with default settings. In addition, we assessed the internal homogeneity of the fibroblast subsets using the Ratio of Global Unshifted Entropy (ROGUE) algorithm (v1.0)^35^ with default parameters. A ROGUE value approaching 1 indicates a relatively homogeneous subset with minimal intra-cluster variation.

The trajectory of fibroblast subsets was inferred by PHATE (v1.0.7)^36^ based on harmony embedding values from the dimensions accounting for 90% of the total variance. To minimize batch effect, RNA velocity was calculated using TFvelo (v1.0)^37^ with the minimum Spearman coefficient = 0.7 based on the dataset of Wirka *et al*.^3^ Pseudotime values and RNA velocity vectors were mapped onto the corresponding cells on the previously generated PHATE plots. The deep learning framework CytoTRACE 2 was used to predict the developmental potential of fibroblast subsets.^38^ As CytoTRACE 2 officially recommended running different batches separately, we analysed the fibroblast subsets individually based on the study they originated from. Trajectory from fibroblast to SMC was inferred using Monocle (v2.30.0).^39^ The pseudotime origin was predicted by CytoTRACE 2. Then we created the object for the proposed pseudotime analysis using the ‘newCellDataSet’ function and then inferred cell differentiation trajectories based on the top 2000 DEGs among cell types selected by ‘differentialGeneTest’ function with batch effect corrected.

### 2.3 Functional and gene signature enrichment analysis

For functional analysis, we performed Gene Ontology analysis, irGSEA,^40^ GSEA,^41^ and PROGENy^42^ in combination with decoupleR.^43^ Gene Ontology analysis of each fibroblast subset was performed with clusterProfiler (v4.10.0),^44^ using the significant DEGs of each fibroblast subset (adjusted *P*-value < 0.05 and an average of log_2_ foldchange > 0.5). irGSEA and GSEA were performed using HALLMARK gene sets and ‘NABA_matrisome’^45^ obtained from the Molecular Signature Database (MSigDB) with default parameters.^46^ The genes used for GSEA analysis were expressed in at least 10% of cells of a given cell type. GseaVis R package (v0.1.0, https://github.com/junjunlab/GseaVis) was used for GSEA result visualization. DecoupleR was used with default parameters.

Gene signature enrichment analysis was calculated using the UCell R package^47^ (v2.6.2) with top DEGs of the cluster.

### 2.4 Experimental animals

For single-cell sequencing experiments, male mice of 8–9 weeks old, deficient for the low-density lipoprotein receptor (*Ldlr^−/−^*), were fed a standard laboratory diet (*n* = 15) or a high cholesterol diet for 16 weeks (HCD, 0.25% cholesterol, 15% cocoa butter, 10% corn starch, 20% casein, 40.5% sucrose, 5.95% cellulose, 824171, Special Diet Services, *n* = 13) to induce atherosclerosis. For primary adventitial fibroblast culture, male C57BL/6J mice were used. For lineage tracing experiments, mice heterozygote for fibroblast Cre driver (*Pdgfra^tm1.1(cre/ERT2)Blh^*/J) and homozygote for tdTomato (*Gt(ROSA)26Sor^tm14(CAG-tdTomato)Hze^*/J)^48^ mice were crossed to wild-type C57/bl6.^49^  *Pdgfra^tm1.1(cre/ERT2)Blh^*/J)x(*Gt(ROSA)26Sor^tm14(CAG-tdTomato)Hze^*/J) mice were crossed more than nine generations onto C57BL/6 background after acquisition from the Jackson Laboratories, prior to experiments.

Male *Pdgfra*-Cre^ERT2^/tdTomato heterozygous mice (age 7–19 weeks) received tamoxifen (0.1 mg/kg/day, Sigma) intraperitoneally for 3 consecutive days to induce Cre recombinase activity according to previous methods.^50^ One to two weeks after tamoxifen, mice were sacrificed to study tdT expression in young control mice (*n* = 5), or injected intravenously with recombinant adeno-associated virus serotype 8 expressing proprotein convertase subtilisin/kexin type 9 (AAV8-PCSK9, 10E11/ms ICGEB Giacca laboratory) to overexpress *Pcsk9* to silence LDL receptor expression and together with a HCD (0.25%, U8954P0296, SAFE) for 10 or 20 weeks induced atherogenesis (*n* = 9, 12 resp.) Aged, control mice received standard diet for 20 weeks and no AAV8-PCSK9 (*n* = 11).

All mice were housed in the Maastricht University laboratory animal facility under standard conditions, in individually ventilated cages (GM500, Tecniplast) with up to five animals per cage, with bedding (corncob, Technilab-BMI) and cage enrichment. Cages were changed weekly, reducing handling of mice during non-intervention periods. All interventions were performed during the light cycle. Mice were euthanized either by an intraperitoneal pentobarbital injection (150 mg/kg), followed by opening of the respiratory cavity, or by gradual fill CO_2_ inhalation at a flow rate corresponding to 30% of the chamber volume per minute standardized using an EZ-DOCK SMARTBOX (EZ systems). Mice were monitored for loss of reflexes and respiration, and the heart was promptly removed upon absence of the toe-pinch reflex. Blood was collected for flow cytometry, the aortic root and brachiocephalic arteries were dissected for histology, and the thoracic aorta was collected for single-cell sequencing.

### 2.5 Single-cell isolation and RNA sequencing of paired mouse adventitia and plaque

Adventitial tissue was microscopically dissected from intima and media of the thoracic aorta (aortic root to diaphragm), and separate fractions were collected in PBS on ice. Tissues from the same fraction of several mice were pooled per diet group and enzymatically digested for 15 min at 37°C using pronase (0.01 g/ml, 10165921001, Roche), collagenase B (0.00284 g/ml, 11088807001, Roche), and DNAse (0.1 mg/ml, 11284932001, Roche). This pronase/collagenase digestion protocol was selected to optimize fibroblast recovery but is less efficient for medial SMC recovery than Liberase/Elastase-based protocols. Cells were passed through a 70-µm cell strainer and subjected to red blood cell lysis (8.4 g/L NH_4_Cl, 0.84 g/L NaHCO_3_ in dH_2_O, pH 7.4). Thereafter, living DAPI-negative cells were sorted on FACS Fusion (BD Biosciences) before scRNA-seq. For scRNA-seq, 4822 cells of adventitia on chow, 7989 cells of adventitia on 16-week HCD, and 3976 cells of the plaque with media were loaded onto a Chromium 10× Genomics controller using the 10× Genomics V3 reagent kit. In short, within gel beads in emulsion, cells were lysed. Thereafter, barcoded oligonucleotides were reverse transcribed followed by clean-up and cDNA amplification. The 10× Genomics Chromium Single-Cell 3′ Library kit was used to generate indexed sequencing libraries. Sequencing was performed using Illumina HiSeq4000. Data are uploaded to Gene Expression Omnibus (GEO; GSE277853, available at time of publication).

### 2.6 ScRNA-seq analysis of paired mouse adventitia and plaque

The 10× Cell Ranger pipeline (v3.0.2) was used to align raw sequencing reads to the mouse reference genome (mm10), and to perform filtering and barcode and unique molecular identifiers (UMI) counting (see Supplementary material online, *Table S2*). Initial quality control was performed by removing cells with expression of less than 200 or more than 6000 genes, with a UMI count lower than 200 or more than 25 000, with a percentage of mitochondrial reads above 10%, and a percentage of haemoglobin gene content above 5%. This yielded a total of 3992 cells adventitia on the chow sample, a total of 5068 cells adventitia on the 16-week HCD sample, and a total of 2764 cells from the intimal + medial + plaque tissue sample. A similar data preprocessing workflow described above was applied and mitochondrial contamination was regressed out during the scaling process. Thirty harmony embeddings were used for clustering (resolution = 0.1, selected using Clustree package) and UMAP. Identified clusters were annotated based on established markers found in the top DEGs of each cluster. Fibroblast subsets were further reclustered using similar methods with a resolution of 0.5. Next, the enrichment of gene signatures of mouse meta-analysed fibroblast subsets on individual cells in fibroblast subsets was calculated. Final fibroblast subsets were annotated based on the UCell enrichment scores along UMAP coordinates and meta-analysed fibroblast subset markers. Monocle (v2.30.0) was used to perform pseudotime analysis on fibroblast subsets using similar parameters mentioned above. The pseudotime origin of fibroblast subsets was set based on the result of mouse meta-analyses.

### 2.7 The distribution analysis of fibroblast subsets

We calculated the ratio of observed to expected cell numbers (*Ro/e*) for each fibroblast subset in different layers and disease status to quantify the layers and disease status preference of each subset.^51^ One subset was identified as being enriched in a specific layer (or disease status) if *Ro/e* > 1.

### 2.8 Meta-analysis of human control and atherosclerotic arteries scRNA data

Raw count matrices of each library from human coronary arteries without macroscopic evidence of atherosclerosis and with atherosclerosis were downloaded from GEO (GSE131778) and Zenodo (doi.org/10.5281/zenodo.6032099).^3,27^ Initial quality control was performed by removing cells with expression of less than 200 or more than 4000 genes, with a UMI count lower than 200 or more than 20000, with a percentage of mitochondrial reads above 10%, and a percentage of haemoglobin gene content above 5%. Doublets were identified and removed by DoubletFinder (v2.0.4).^52^ The integration tool ‘Harmony’ failed to cluster cells of the same type together (data not shown), which suggests an overcorrection of batch effects. Therefore, we used the Seurat CCA integration method with SCTransform, the recommended normalization approach for CCA. Data were then normalized using SCTransform with parameter (vst.flavor = v2). Integration with 3000 most highly variable genes selected by SelectIntegrationFeatures function was done using the PrepSCTIntegration, FindIntegrationAnchors, and IntegrateData functions. The batch-corrected expression matrix was then used for PCA dimensionality reduction, followed by cell clustering (30 PCs, resolution 0.24, selected using the Clustree package) UMAP visualization. DEGs of each cluster were identified using matrices normalized with the ‘NormalizeData’ function. Identified clusters were then annotated to different cell types based on established markers found in the DEGs. Fibroblast subsets were then reclustered using a similar workflow with 2000 of the most highly variable genes and a resolution of 0.18, which was selected using the Clustree package.

### 2.9 Integration of meta-analysed mouse and human fibroblasts

To integrate meta-analysed mouse and human fibroblast subsets, we used SATURN,^53^ a deep learning method for learning universal cell embeddings that encodes genes’ biological properties using protein language models. The analysis was performed using default settings with ESM2, a large protein embedding language model.

### 2.10 Correlations with human atherosclerotic plaque traits

The AtheroMAAS cohort plaques were stratified based on the presence/absence of plaque with intraplaque haemorrhage (IPH), an established hallmark of plaque stability and event risk.^26,54^ Twenty-seven IPH and 16 non-IPH snap-frozen plaque samples from carotid endarterectomy were used for Illumina microarray transcriptomics analysis. In the 86 adjacent formalin-fixed paraffin embedded segments, plaque traits were quantified: plaque area; content of necrosis, collagen, and calcification; vascular cell-type densities; microvascular and lymphatic densities. Further details and information regarding sample collection, RNA extraction, data processing and normalization, and assessment of plaque traits have been previously described.^26,54^

We then calculated Pearson correlations of the top 50 DEGs per fibroblast subset with plaque traits in 43 human carotid plaque segments. Significance was assessed by positive and negative correlations and the imbalance thereof, which was defined as the sum of positive correlations minus the sum of absolute values of negative correlations. Furthermore, correlation skewness was compared between DEGs and a random gene set containing a similar number of genes. The permutation test was performed 100 000 times, and the *P*-value was the frequency of the random gene sets that have higher correlation skewness than the DEG sets.

### 2.11 Immunohistochemical and immunofluorescent staining and visualization

Murine aortic roots were dissected and fixed in 1% paraformaldehyde overnight. Brachiocephalic arteries were fixed in 4% paraformaldehyde overnight. Subsequently, tissue was paraffin-embedded and serially sectioned (4 µm). Thereafter, aortic root tissue was stained and subjected to heat-induced epitope retrieval at low pH (EnVision FLEX Target Retrieval Solution, pH low [#K8005] or high pH (Agilent Dako, Santa Clara, USA, [#GV800]) depending on the antibody. Sections were stained for tdTomato alone or followed by antibodies for cell identity (SM22α, MYH11 for SMCs, Pecam1/CD31 for ECs, CD68 for leukocytes/macrophages), fibroblast subset 2 (UDP-glucose 6-dehydrogenase [UGDH]), or for proliferation (PCNA, abcam, #92552) according to Supplementary material online, Table  *S3*. Cells positive or negative for tdT were then termed *Pdgfra* Lin^+^ and *Pdgfra* Lin^−^ cells.

For immunohistochemistry, primary antibodies were resolved through alkaline phosphatase or hydrogen peroxidase coupled tertiary kits Via ABC-AP or ABC-HRP Elite (AK-5000 Vectastain, #30085, Vector Laboratories). Colorimetric detection was done with Vector Red (Vector SK-5100) or 3,3′-diaminobenzidine (DAB, Agilent K346811-2), respectively. Following counterstaining with haematoxylin, cubes were made using the PerkinElmer Nuance bright-field microscope. Individual colours were separated according to previously described protocols.^7,55,56^ Briefly, images were made through PLAN APO ×20/0.70 and HC PLAN APO ×40/0.75 water objectives, and an HXC PLAN APO ×63/×100/1.40–0.70 oil immersion objective. Cubes were made using a Nuance Multispectral Imaging System (PerkinElmer, Hopkinton, MA, USA) with version 3.0.2 software. Nuance microscopy registers the spectrum of light emitted by a specimen when excited in a narrow wavelength range. We applied excitation wavelengths between 420 and 460 nm, while emission was evaluated through a long-pass filter. A spectrum was obtained in ascending steps of 20 nm, allowing to separate the different colorimetric precipitates as they are visible at different wavelengths. Pseudo-colours were assigned to unmixed images, and composite images showing co-localization were generated with the Nuance 3.0 software. Quantitative assessment of co-localization and pixel-based measurement of the individual markers per microscopic field was done with the same software. Co-localizing pixels were quantified per region of interest (ROI) in the aortic root tunica adventitia, tunica media and tunica intima/plaque, or in the plaque cap. The fibrous cap area was identified on α-SMA stained section. The fibrous cap was defined as the α-SMA^+^ region overlaying the necrotic core, spanning the luminal surface of the lesion only, excluding adjacent fibrous cap shoulder regions. The cap area ROI was manually delineated using the Nuance software based on α-SMA positivity and structural integrity. ROIs were automatically segmented using a thresholding algorithm, followed by manual verification to exclude artefacts and background staining. Testing measurements were performed by blinded investigators after reproducibility was ensured with an experienced vascular pathologist (Marion J. Gijbels). Data were presented as an average per mouse.

For immunofluorescent staining, paraffin slides were deparaffinized, and heat-induced antigen retrieval was performed at low pH (Dako). Autofluorescence was quenched in quenching buffer (26.4 mM NaOH in PBS + 4.6% H_2_O_2_) using intense white light exposure for 30 min. Subsequently, non-specific binding was blocked using 2.5% normal horse serum for 30 min. Next, primary antibodies (murine Pecam1/CD31, tdTomato, human PECAM1, human PI16, or human PDGFRA) were incubated in 1% BSA/PBS overnight at 4°C according to Supplementary material online, Table  *S3*.

Secondary antibodies coupled to Alexa Fluor 488 (1:50, Invitrogen) or Alexa Fluor 680 (1:200, Invitrogen) were incubated at room temperature for 30 min. Nuclei were stained with DAPI. Images were acquired using a Leica SP8 confocal microscope with either Fluotar VISIR 25×/0.95 or HC PL APO 86×/1.20 water immersion objectives. Z-stacks (0.6 µm step size) were used to produce orthogonal sections within the Leica Application Suite X (Leica Microsystems).

### 2.12 Cell culture and stimulation

Human aortic adventitial fibroblasts were derived from three donors: two commercially obtained donors (Lonza, CC-7014; 51-year-old female and 63-year-old male; batches 580579 and 23TL104316, respectively) and one donor from adventitial outgrowth of a human aortic specimen (41-year-old female). Cell type purity was validated in our lab (see Supplementary material online, *Figure S1*). Human aortic adventitial fibroblasts were cultured with stromal cell growth medium (SCGM) containing stromal cell basal medium supplemented with 5% fetal bovine serum (FBS), basic fibroblast growth factor, and insulin (all included in CC-3205, Lonza Company). Human macrophages were derived from the THP-1 human monocytic leukaemia cell line (ATCC, TIB-202). THP-1 cells were cultured in RPMI medium (Gibco #72400-021) supplemented with 10% FBS (Serana #S-FBS-SA-015) and 1% penicillin–streptomycin (Gibco, 15070-063) at 37°C in a humidified atmosphere with 5% CO_2_. Differentiation into macrophages was induced by treating cells with 200 nM phorbol 12-myristate 13-acetate (PMA, Sigma, P1585) for 48 h, followed by incubation in fresh medium without PMA for 24 h. The human microvascular EC line HMEC-1 (ATCC, CRL-3243), originally isolated from foreskin, was cultured in the same RPMI medium as above. Human vascular smooth muscle cells (HSMCs)^57^ were cultured in DMEM medium (Invitrogen, 31966047) supplemented with 10% FBS (Serana, S-FBS-CO-015) and 1% penicillin–streptomycin (Gibco, 15070-063) at 37°C with 5% CO_2_.

Human aortic adventitial fibroblasts were maintained at 37°C with 5% CO_2_, and the growth medium was changed the day after seeding and every other day thereafter. Cells (3 × 10^4^) were seeded in five replicate wells in a 24-well plate and incubated overnight. After starvation with 0.5% FBS SCGM medium for 24 h, the cells were subjected to 0% FBS starvation, 25 µg/ml oxidized LDL (prepared as described before^58^), 0.2 µg/ml IFNy (R&D, 285-IF-100), 50 ng/ml TGFb1 (PeproTech, 100-21), 50 ng/ml TGFb2 (R&D, 302-B2-010), 50 ng/ml TGFb3 (Immunotools, 11344483), 0.2 µg/ml IL1B (PeproTech, 200-01B), or 25 µM H_2_O_2_ for 24 h or 1% oxygen (hypoxia) for 72 h prior to further experimentation.

### 2.13 Flow cytometry

Flow cytometry was performed using circulating cells, adventitia, and media with intima of *Pdgfra*-Cre^ERT2^/tdTomato mice on chow diet, as well as cultured human aorta fibroblasts. To quantify absolute leukocyte counts as previously done,^59^ whole blood was added to BD Trucount Absolute Counting Tubes (340334) containing Fc receptor block (CD16/32, eBioscience 14-0161-82). After incubation with CD45 (CD45 PerCP, Biolegend, 103130), the samples were measured in erythrocyte lysis buffer on a Cytek Aurora flow cytometer (4L:6UV-16V-14B-8R, Cytek Biosciences) and analysed using SpectroFlo software (Cytek Biosciences). Single cells were isolated from thoracic arch adventitia or media (with intima), labelled with antibodies for CD45, PDGFRA, and CDH5, and gated as described.^7^ Human aorta fibroblasts were dissociated, and transferred from a 24-well plate to a 96-well V-bottom plate. These cells were then incubated on ice for 10 min with FcR block (1:100, 14-0161-85, ThermoFisher) and Fixable Viability Dye eFluo780 (1:1000, 65-0865-14, ThermoFisher) in PBS. Following this, Alexa Fluor 647 conjugated anti-CDH5 (1:200, 561567, BD biosciences) was added in FACS buffer (PBS 0.5% BSA, 0.1% NaN3), and the cells were incubated on ice for 30 min. Cells were then washed, resuspended in FACS buffer, and immediately measured on a Cytek Aurora flow cytometer with SpectroFlo software.

### 2.14 RNA isolation, cDNA synthesis, and RT-qPCRs

RNA was isolated using QIAzol reagent (79306, Qiagen), followed by the addition of chloroform with subsequent phase separation. This was performed according to manufacturer’s protocol. RNA concentrations were determined using a Nanodrop 2000 (Thermo Fisher Scientific), and cDNA was synthesized using the iScript Select cDNA Synthesis Kit (1708891, Bio-Rad). RT-qPCRs were performed using iQ SYBR Green Supermix (170885, Bio-Rad), 10 ng cDNA input, and specific primer sets (see Supplementary material online, *Table S4*).

### 2.15 Statistical analyses

Measurements were taken from independent samples (mice/culture wells with cells), and no repeated measurements were performed. Graphs are represented as mean ± standard error of the mean (SEM). All results were analysed with GraphPad Prism version 10 (GraphPad Software Inc.). All statistical tests were two-sided. ROUT outlier analysis was performed, and any outlier was excluded. Thereafter, normality (Shapiro–Wilk) and equal-variance (*F*-test, two-sided) tests were performed, and the corresponding parametric (*T*-test or ANOVA, as appropriate, with *post hoc* Tukey tests) or non-parametric testing were applied (Mann–Whitney or Kruskal–Wallis). All *P* values for statistical tests examining more than two groups were adjusted for multiple comparisons (Tukey or Dunn’s). Correlations were analysed by Pearson’s *r*. *P < 0.05, **P < 0.01 and ***P < 0.001, ****P < 0.0001.

## 3. Results

### 3.1 The majority of fibroblasts in murine atherosclerosis do not derive from *Myh11* lineage-traced smooth muscle cells

While fibroblasts have been identified based on annotations of RNA transcriptome in human and mouse atherosclerosis using scRNA-seq, we lack a precise understanding of their identity, origin, and fate. First, a shared identity with and/or origin from SMCs was explored because of previously observed transitions of *Myh11*-lineage SMCs into fibroblast-like cells in atherosclerosis.^3^ We used a meta-analysis of murine scRNA-seq data including 70 590 cells from control aorta and atherosclerotic plaques of myosin heavy chain 11 (*Myh11*) lineage reporter mice on chow and HCD to identify cells of SMC lineage in fibroblast populations (*Figure 1A*; Supplementary material online, *Figure S2A*–*E*; Supplementary material online, Table  *S1*).^2,3,6,28^ Cells were annotated based on expression of previously established markers, i.e. *Myh11*, Calponin 1 (*Cnn1*), and Transgelin (*Tagln*) for SMCs; vascular adhesion molecule 1 (*Vcam1*) and TNF Receptor Superfamily Member 11b (*Tnfrsf11b*) for fibromyocytes; and Collagen 2a1 (*Col2a1*) and aggrecan (*Acan*) for fibrochondrocytes/chondromyocytes. Fibroblasts were defined by high expression of established fibroblast markers, including *Pdgfra* and dipeptidase 1 *(Dpep1)*, with low expression of canonical SMC, endothelial, or immune-cell markers.^2,3,6,7,28,60^ In this meta-analysis, the marker genes peptidase inhibitor 16 (*Pi16*), cytoglobin B (*Cygb*), and Serpin F1 (*Serpinf1*) were most specific for fibroblasts with negligible expression of other cell type markers (*Figure 1B*). *Myh11* lineage reporter information from both apolipoprotein E knockout (*ApoE*^−/−^) and low-density lipoprotein receptor knockout (*Ldlr*^−*/*−^) mice showed the expected *Myh11* origin of SMCs, and revealed a dual lineage composition of fibroblasts, dominated by *Myh11*-lineage-negative cells. A total of 94.1% of fibroblasts did not derive from the *Myh11* lineage, while 5.9% were positive for the *Myh11* lineage reporter (*Figure 1C*; Supplementary material online, *Figure S2F*). The acquisition of *Acta2* expression by fibroblasts was traditionally assigned to a myofibroblast phenotype.^12^  *Acta2* was, however, only detected in 33% of *Myh11*-lineage-negative (*Myh11* Lin^−^) fibroblasts, in contrast to 87% of *Myh11*-lineage-positive (*Myh11* Lin^+^) fibroblasts (see Supplementary material online, *Table S5*). Additionally, *Myh11* Lin^+^ fibroblasts showed higher *Acta2* expression levels than *Myh11* Lin^−^ fibroblasts (see Supplementary material online, *Figure S2G*), consistent with a stronger SMC-associated transcriptional programme. Thus, *Acta2* expression alone does not distinguish myofibroblasts from *Myh11-*derived fibromyocytes.

**Figure 1:**
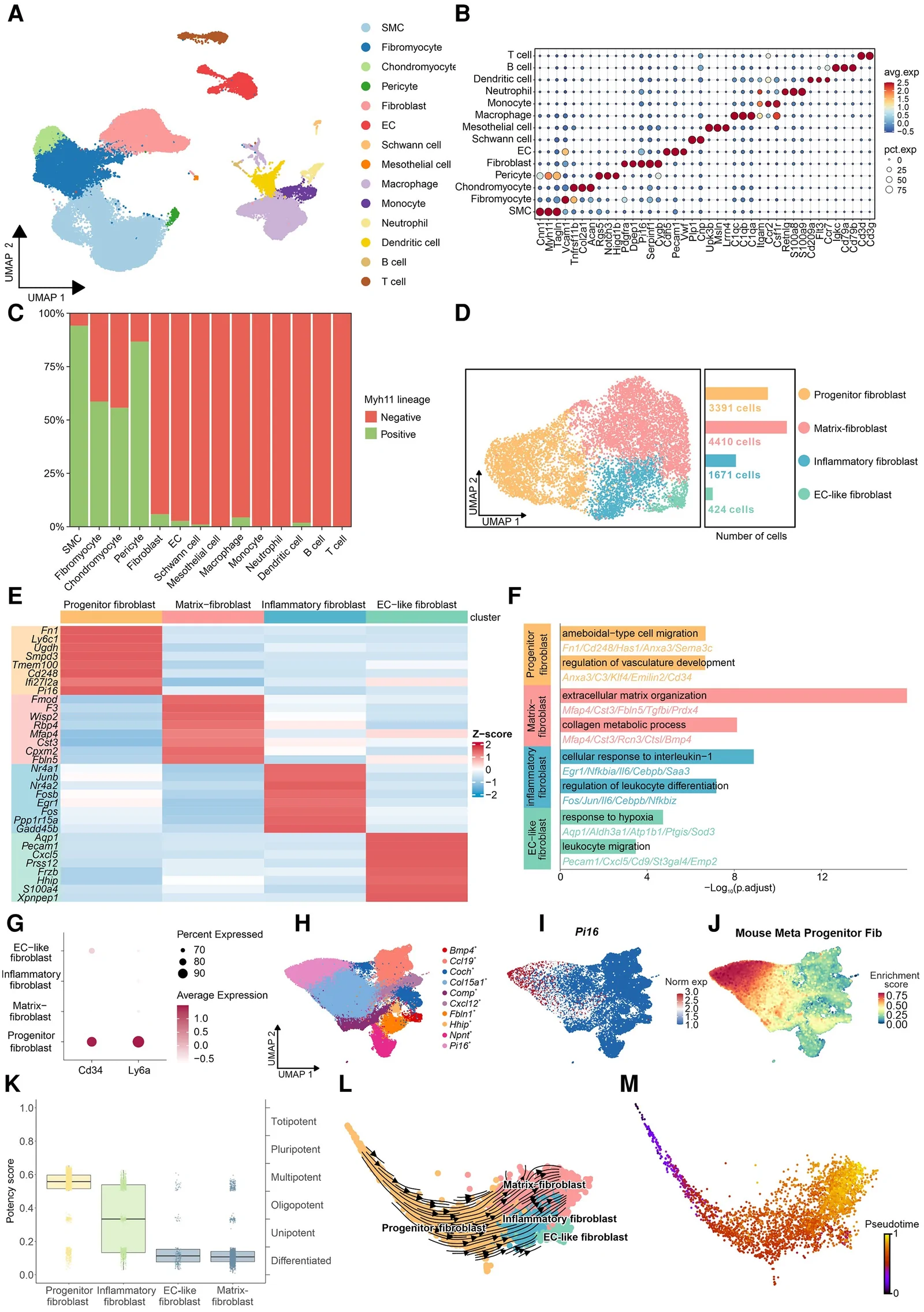
The majority of plaque fibroblasts derive from non-MYH11 origin and the adventitia. (*A*) UMAP embeddings of 70 590 single cells from 38 sequencing libraries across 4 studies are colour coded by cell types. (*B*) Dot plot of canonical marker genes of cell types. Dot diameter represents the percentage of cells in certain cell types expressing corresponding genes. (*C*) Proportions of *Myh11 Lin^+^* cells (green) and *Myh11 Lin*^−^ cells (red) across annotated cell types in the combined Pan et al. (*Ldlr^−^/^−^*) and Wirka et al. (*ApoE^−^/^−^*) datasets. Fibroblasts mostly derived from Myh11-lineage-negative cells. (*D*) UMAP embeddings and cell numbers of 4 fibroblast subsets. (*E*) Heatmap showing the average expression of top 8 marker genes in 4 fibroblast subsets. (*F*) Key functions of 4 fibroblast subsets based on GO enrichment analysis. (*G*) The expression of *Cd34* and *Ly6a* within 4 fibroblast subsets. Progenitor fibroblast had the highest expression of these two genes. (*H*) UMAP visualization of 120 583 cells in the fibroblast cross-organs atlas from healthy-state mice. (*I*) FeaturePlot showing expression of *Pi16* within fibroblast subsets. (*J*) UCell enrichment of the top 20 DEGs within meta-analysed progenitor fibroblast compared with other fibroblast subsets on fibroblast cross-organs atlas from healthy-state mice. The progenitor fibroblast shows a high similarity with the *Pi16^+^* cluster which was the original fibroblast. UCell scores were calculated using the Mann–Whitney *U* statistics. (*K*) The cellular potency category of 4 fibroblast subsets in published scRNA data from the mouse aortic root (Wirka et al.)^3^ predicted by CytoTRACE 2. Progenitor fibroblast exhibited the highest developmental potential, followed by inflammatory fibroblast, EC-like fibroblast and matrix-fibroblast. (*L*) PHATE embeddings of fibroblast subsets in published scRNA data from the mouse aortic root (Wirka et al.) with the arrows showing RNA velocities. Progenitor fibroblast, as the start point, directly developed into a matrix-fibroblast or developed into an inflammatory fibroblast first. Subsequently, the inflammatory fibroblast further developed into a matrix-fibroblast or EC-like fibroblast. (*M*) PHATE embeddings of fibroblast subsets in published scRNA data from the mouse aortic root (Wirka et al.) coloured by pseudotime.

In addition to a SMC origin of fibroblasts, ECs have also been reported to transition into fibroblast-like cells via EndoMT.^8,19^ The study by Alencar *et al*.^28^ also included scRNA-seq data from the EC lineage reporter VE-Cadherin (*Cdh5*). None of the cells in these samples could be annotated as fibroblasts, suggesting ECs are not the main origin of fibroblasts in arteries of atherosclerotic mice (see Supplementary material online, *Figure S2H*).

### 3.2 ScRNA-seq predicts one fibroblast subset as the origin of plaque fibroblasts

The origin of the *Myh11* Lin*^−^* fibroblasts was studied further to reveal more details on their cell identity and origin. Sub-clustering of these 9896 cells revealed four distinct fibroblast subsets marked by several DEGs and putative functions predicted by Gene Ontology, and gene set enrichment analysis (*Figure 1D–F*; Supplementary material online, *Figures S3A*–*C* and*S4A* and *B*; Supplementary material online, *Table S6*). Ratio of Global Unshifted Entropy (ROGUE) analysis further supported the robustness of this subset classification, with high ROGUE values across all four fibroblast subsets indicating high internal homogeneity rather than unstable clustering (see Supplementary material online, *Figure S3C*). Although all fibroblast subsets expressed matrix genes as expected, one was identified as the main subset involved in matrix and collagen metabolism and thus termed ‘matrix-fibroblasts’ (*Figure 1F*; Supplementary material online, *Figure S4A* and *B*). Differential genes for a second fibroblast subset suggested involvement in inflammatory responses and leukocyte differentiation and this subset was thus termed ‘inflammatory fibroblasts’. For the third fibroblast subset, genes annotated were associated with the response to hypoxia, and angiogenesis, and included EC marker genes such as *Pecam1*. Hence, cells in this subset were termed ‘EC-like fibroblasts’ (*Figure 1E* and *F*; Supplementary material online, *Figure S4A* and *B*). The fourth fibroblast subset expressed the progenitor markers *Ly6a* and *Cd34* (*Figure 1E, G*). This fibroblast subset was thus renamed to ‘progenitor fibroblasts’ and was predicted to regulate vasculature development (*Figure 1F*). Its functional annotation and gene signature were highly comparable to the transcriptome of cluster of differentiation 55 (*Cd55*)^+^ fibroblasts isolated from the adventitia of healthy, young wild-type mice we described before, such as *Ugdh* and *Cd248* (see Supplementary material online, *Figure S4C*).^7^ Importantly, CD55^+^PDGFRA^+^ cells were not only detected in the adventitia, but also in the murine and human atherosclerotic plaque using flow cytometry and immunohistochemistry.^7^ Progenitor fibroblasts also showed elevated expression of *Ugdh*, *Pi16*, and *Ly6c1*, compared to other plaque fibroblasts, resembling a ubiquitous, adventitial fibroblast, recently identified in a transcriptome atlas of 120 583 fibroblasts across organs from healthy mice (*Figure 1H* and *I*).^61^ These ubiquitous *Ly6c1^+^Pi16^+^* fibroblasts were universally present across organs, lining the vasculature as confirmed by RNAscope-based localization and a dermopontin lineage reporter mouse model.^61^ Additionally, *Ly6c1^+^Pi16^+^* fibroblasts were the observed source for differentiation into more specialized, organ-specific fibroblasts. Progenitor plaque fibroblasts were indeed transcriptionally comparable to the ubiquitous, adventitial *Ly6c1^+^Pi16^+^* fibroblasts as the top 20 gene signature score of progenitor fibroblasts mapped to *Pi16^+^* fibroblasts of the cross-organ atlas (*Figure 1H–J*).

These data point towards progenitor fibroblasts stemming from the adventitia and being the origin of differentiation for plaque fibroblasts. We used a deep learning framework to predict the development potential of aortic fibroblast subsets based on gene expression signatures of *ApoE*^−/−^ and *Ldlr*^−/−^ mice on chow and HCD.^2,3^ Progenitor fibroblasts were predicted to be multipotent, while inflammatory fibroblasts showed a mixed differentiation potential, and matrix-fibroblasts and EC-like fibroblasts a fully differentiated state (*Figure 1K*; Supplementary material online, *Figure S4D*). Notably, the potency score of progenitor fibroblasts did not reach a pluripotent state, but clearly multipotent assignment across datasets. Expression of traditional mesenchymal stem cell markers was low and infrequent, suggesting progenitor fibroblasts do not fully resemble resident mesenchymal stem cells (see Supplementary material online, *Figure S4E*).^62^ Nevertheless, RNA velocity analysis of plaque fibroblasts in our plaque meta-analysis also supported the progenitor fibroblast subset as the ‘start point’ for differentiation into the three other fibroblast subsets (*Figure 1L* and *M*). Thus, progenitor fibroblasts seem a probable origin of more specified fibroblast subsets, likely stemming from the adventitia.

### 3.3 Plaque fibroblasts originate from adventitial progenitor fibroblasts

Still, the unambiguous distinction between adventitial and plaque fibroblast and their origin required further scrutiny, as datasets compiled in our plaque meta-analysis did not include the adventitia or failed to mention their sampling approach towards inclusion/exclusion of the adventitia (see Supplementary material online, *Table S1*). Thus, we isolated independent preparations of adventitia and plaque with media from *Ldlr*^−/−^ mice on chow or HCD for scRNA-seq analysis to further substantiate the adventitial origin of plaque fibroblasts (*Figure 2A*). After data integration, all major cell types, including endothelial, mesenchymal and immune cells, were detected and annotated in line with the differential cell type markers from the meta-analysis (*Figure 2B–D*; Supplementary material online, *Figure S5A* and *B*). Fibroblast subtype analysis identified 6 transcriptionally distinct subsets in this integrated dataset of plaque and adventitia (*Figure 2E*; Supplementary material online, *Figure S5C* and *D*). Four of the six subsets matched the four plaque subsets from the meta-analysis as evidenced by shared expression of differentially expressed markers and enrichment of signature expression scores while two subsets were exclusive to this dataset and not identified before (*Col5a3*^+^ and *Pou2f2*^+^) (*Figures 1D* and *E and 2F–J*). In addition, the signature of genes differentially expressed by fibroblasts from the adventitial samples was enriched in the progenitor fibroblast subset of the plaque meta-analysis (*Figure 2K*). Conversely, the plaque fibroblast gene signature was enriched in matrix-fibroblasts, inflammatory fibroblasts and EC-like fibroblasts of the meta-analysis (*Figure 2K*). Thus, we annotated the fibroblast subsets of the adventitia-plaque dataset as progenitor fibroblasts, matrix-fibroblasts, inflammatory fibroblasts, EC-like, *Col5a3*^+^, and *Pou2f2*^+^ fibroblasts accordingly. The progenitor fibroblasts were preferentially located in the adventitia compared to plaque, while matrix- and inflammatory fibroblasts were enriched in plaques compared to adventitia (*Figure 2E*; Supplementary material online, *Figure S5E*). Interestingly, the *Col5a3^+^* fibroblast subset was nearly absent in plaques and enriched in cells isolated from the adventitia (*Figure 2E*; Supplementary material online, *Figure S5E*). This may explain why *Col5a3^+^* fibroblasts were not observed in the meta-analysis. The newly discovered *Pou2f2^+^* subset consisted only of 71 cells and was present among cells isolated from the adventitia and plaques. This small subset was not observed in the large meta-analysis, despite inclusion of nearly 71 000 cells nor in our prior adventitial dataset of the adventitia dissected from arteries of healthy, C57BL/6 mice,^7^ and does not seem reproducible. For other subsets, presence or absence in the adventitia was in line with prior adventitial data.^7^ There, we identified *Cd55^+^*, *Lox^+^* and *Cxcl14^+^* fibroblasts,^7^ which show enrichment of the gene signatures of progenitor fibroblasts, matrix-fibroblasts and *Col5a3*^+^ fibroblasts, respectively (see Supplementary material online, *Figures S4C* and *S5F* and *G*). Inflammatory and EC-like gene sets do not show significant enrichment in the fibroblast subsets of healthy mice, fitting their disease association (see Supplementary material online, *Figure S5H* and *I*). In line, the progenitor and inflammatory subset proportions within all fibroblasts expanded with disease progression in plaques, while the matrix fibroblast proportion decreased (see Supplementary material online, *Figure S5J* and *K*). Finally, pseudotime analysis of the new parallel plaque-adventitia dataset also supported the differentiation trajectory of fibroblast subsets with progenitor fibroblasts as the start point (*Figure 2L–N*).

**Figure 2:**
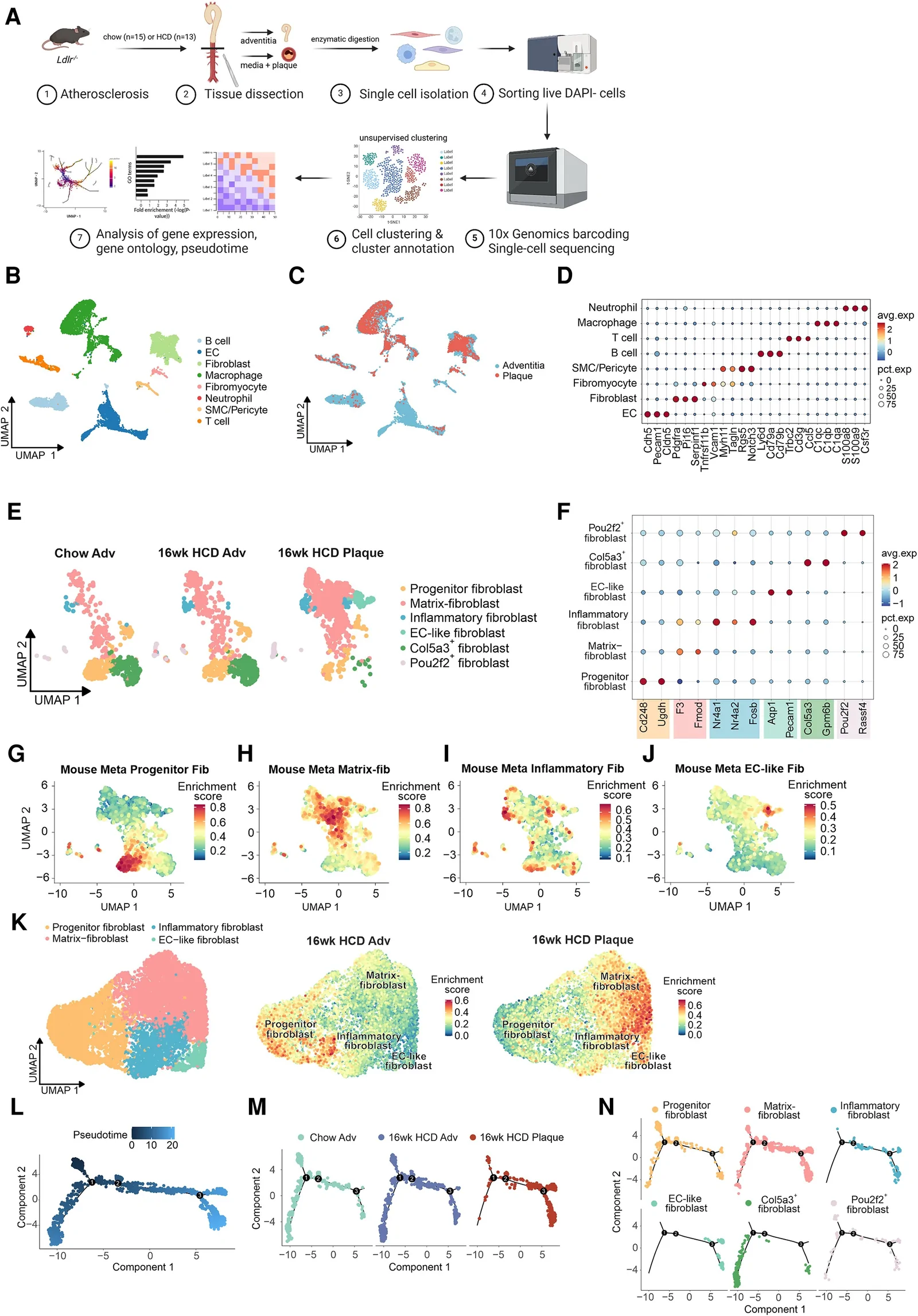
Single-cell sequencing identifies adventitia as the origin of plaque fibroblasts. (*A*) Setup of *Ldlr*^−*/*−^ mouse experiment as performed for scRNA-Seq of adventitia and plaque with media on chow and HCD. (*B*) UMAP embeddings of 11 824 single cells from adventitia and plaque with media of LDLR-Ko mice on chow and HCD. (*C*) UMAP visualization of cells colour-coded by the layers. (*D*) Dot plot of canonical marker genes of cell types. Dot size represents the portion of cells expressing the gene. (*E*) UMAP embeddings of fibroblast subsets split by each sample. Annotations were defined using UCell scores and differentially expressed marker genes of meta-analysed fibroblast subsets. (*F*) Dot plot of top marker genes from meta-analysed fibroblast subsets. *G–J*. UCell enrichment of the top 50 differentially expressed genes within meta-analysed fibroblast subsets compared with other fibroblast subsets on adventitia and plaque fibroblast subsets. (*K*) UCell enrichment of the top 20 differential expression genes of adventitia vs. plaque with 16-week HCD on meta-analysed fibroblast subsets. Meta-analysed progenitor fibroblast mainly derived from adventitial while matrix-fibroblast, inflammatory fibroblast and EC-like fibroblast accumulated in the plaque. *L–N*. Monocle 2 trajectory analysis displayed by pseudotime, samples and fibroblast subsets. The development of progenitor fibroblast into matrix-fibroblast or inflammatory fibroblast was a shared trajectory among adventitia and plaque, while the development of progenitor fibroblast into Col5a3^+^ fibroblast was a specific trajectory for the adventitia.

Taken together, two independent scRNA-seq meta-analyses of 70 590 plaque cells and 120 583 organ fibroblasts, with additional libraries of discrete preparations of murine adventitia and plaques, provide strong support for an adventitial and progenitor fibroblast subset origin for plaque fibroblasts, in addition to a smaller contribution by *Myh11* Lin^+^ SMC.

### 3.4 Plaque fibroblasts derived from *Pdgfra* Lin^+^ cells

While these integrative analyses form a substantive foundation to elucidate the origin of plaque fibroblasts, *in vivo* lineage tracing is required to unequivocally map the origin of cells. Previously, we identified *Pdgfra* as a specific fibroblast marker in healthy human and murine vascular adventitia, both at the RNA and protein levels.^7^ In plaques, however, endogenous *Pdgfra* mRNA is predominantly expressed by fibroblasts, and by *Myh11* Lin^−^ cells, but not exclusively (*Figure 3A* and *B*).^7^ Therefore, *Pdgfra*-Cre^ERT2^*-Ai14tdTomato* mice were used to label *Pdgfra* Lin^+^ cells by tdTomato (tdT) expression upon tamoxifen-mediated induction of the CRE recombinase in young animals on a standard diet (*Figure 3C*). The aortic root of young animals on a standard diet showed a predominant adventitial localization of tdT signal (2.1%), while small fractions of the medial (0.05%) and intimal area (0.3%) were also *Pdgfra* Lin^+^/tdT positive (*Figure 3D–F*). A similar pattern, albeit less frequent, was observed in brachiocephalic arteries (*Figure 3G*). This was unlike antibody detection of endogenous PDGFRA.^7^ Hence, as an orthogonal assay, flow cytometry data was performed and showed that 38% of adventitial tdT^+^ cells in the thoracic aorta were co-expressing endogenous PDGFRA, and 0.45% of intimal CDH5^+^ cells co-expressed tdT (*Figure 3H* and *I*). Further immunohistochemical co-localization of tdT with PECAM1 and SM22α expression showed that 1.9% of PECAM1-stained areas, and 1% of SM22α-stained areas also showed tdT expression (*Figure 3J* and *K*) in the aortic root, while co-localization of PECAM1 was less apparent in the brachiocephalic artery, and abdominal aorta, carotid artery and femoral artery (see Supplementary material online, *Figure S6A* and *B*). This protein data is in line with 5% of cells in the EC and SMC clusters of the meta-analysis expressing very low levels of Pdgfra mRNA (15–25× lower, *Figure 1B*). Of note, tdT expression in circulating CD45^+^ immune cells was absent in animals with positive tdT signals from adventitial cells (*Figure 3L* and *M*).

**Figure 3:**
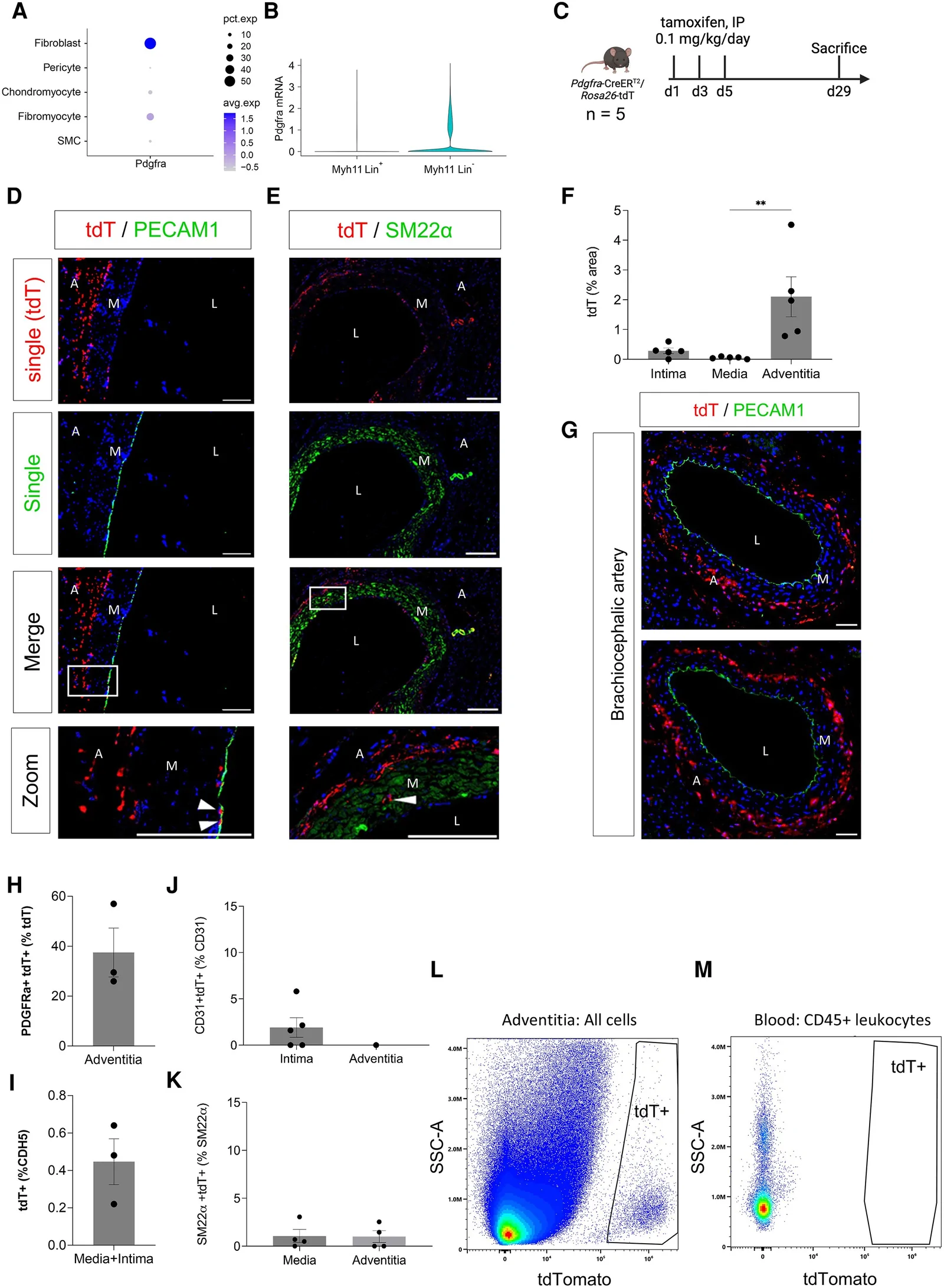
Expression patterns of tdT by platelet-derived growth factor receptor alpha (Pdgfra) lineage-traced cells in young, normocholesterolemic mice. (*A*) Dot plot of *Pdgfra* mRNA in cell types of UMAP from meta-analysis in *Figure 1*. (*B*) Violin plot of *Pdgfra* mRNA in *Myh11* Lin*^+^* cells and *Myh11* Lin^−^ cells of UMAP from meta-analysis. (*C*) Cartoon depicting workflow of *in vivo* studies. (*D*) Single and merged representative immunohistochemical staining of tdTomato (red) and endothelial marker platelet endothelial cell adhesion molecule (PECAM1; green), also known as cluster of differentiation 31 (CD31), shows occasional co-localization (highlighted by arrowheads) across aortic roots of *Pdgfra*-Cre^ERT2^/Rosa26-tdT mice (*n* = 5). (*E*) Single and merged representative immunohistochemical staining of tdTomato (red) and Smooth muscle protein 22-alpha (SM22α; green) shows minimal co-localization (highlighted by arrowhead) across aortic roots of *Pdgfra*-Cre^ERT2^/Rosa26-tdT mice (*n* = 4). (*F*) Quantification of tdT as a percentage of area in intima, media and adventitia (Kruskal–Wallis test, *n* = 5). (*G*) Representative immunohistochemical staining of tdTomato (red) and PECAM1 (green) shows minimal co-localization (yellow) across brachiocephalic arteries of *Pdgfra*-Cre^ERT2^/Rosa26-tdT mice (*n* = 5). *H–I*. Flow cytometry data depicting the percentage of tdT cells co-expressing PDGFRA or CDH5 in *Pdgfra*-Cre^ERT2^/Rosa26-tdT mice (*n* = 3 pools of 2 mice, 6 mice in total). (*J*) Analysis of tdT ^+^PECAM1^+^ area as percentage of PECAM1^+^ area in the intima and adventitia of *Figure 3D* (Mean ± SEM, Statistical significance was determined using a Kruskal–Wallis test), *K*. and the SM22α^+^tdT^+^ area as percentage of SM22α^+^ area in media and adventitia are quantified across aortic roots of *Pdgfra*-Cre^ERT2^/Rosa26-tdT mice in *Figure 3E* (Mean ± SEM, Statistical significance was determined using a Kruskal–Wallis test). (*L*) Scatter plot from flow cytometry experiment showing tdT positive cells in cells isolated from the adventitia of *Pdgfra*-Cre^ERT2^/Rosa26-tdT mice injected with tamoxifen (*n* = 3 pools of 3 mice, 9 mice in total). (*M*) Scatter plot from flow cytometry experiment showing that circulating inflammatory immune cells lack tdT expression in the blood of *Pdgfra*-Cre^ERT2^/Rosa26-tdT mice injected with tamoxifen (*n* = 3 pools of 3 mice, 9 mice in total). Scale bars 100 µm. L; lumen, M; media, A; adventitia, P; plaque. *P < 0.05, **P < 0.01.

To demonstrate that *Pdgfra* Lin^+^ cells were the origin of plaque fibroblasts, tdT expression was induced before induction of atherosclerosis by AAV-mediated PCSK9 overexpression and a high-cholesterol diet (*Figure 4A*). The expression of tdT was only detected in the aortic wall after tamoxifen injection, not in *Pdgfra-Cre^ERT2^-Ai14tdTomato* mice injected with vehicle (*Figure 4B–D*). *Pdgfra*-derived tdT^+^ expression was already detected in fatty streaks of mice on a chow diet for 20 weeks and increased in early and advanced atherosclerotic plaques of the aortic root after 10 or 20 weeks of HCD (*Figure 4E–K*). When normalized to total plaque area, tdT^+^ signal accounted for approximately 5% of the plaque area. The tdT^+^ plaque area correlated with plaque area, necrosis, and collagen content, but not with CD68, PECAM1 or MYH11 content (see Supplementary material online, *Figure S6C*–*H*). In the plaques, spindle-shaped tdT^+^ cells were detected in the cap area, in parallel orientation to the lumen, and alongside the necrotic core pointing towards the lumen (*Figure 4G*). PCNA^+^, proliferating *Pdgfra*-derived tdT^+^ cells are observed in the plaque and adventitia, but not in the media and increase with plaque development (*Figure 4L–R*). As medial tdt^+^ content transiently increased at 10 weeks (*Figure 4I*), without any medial proliferation (*Figure 4N*), this supports the migration of tdT^+^ cells from the adventitia via the media into the plaque. The increase in tdT^+^ fibroblast area in the plaque likely results from migration and local proliferation.

**Figure 4:**
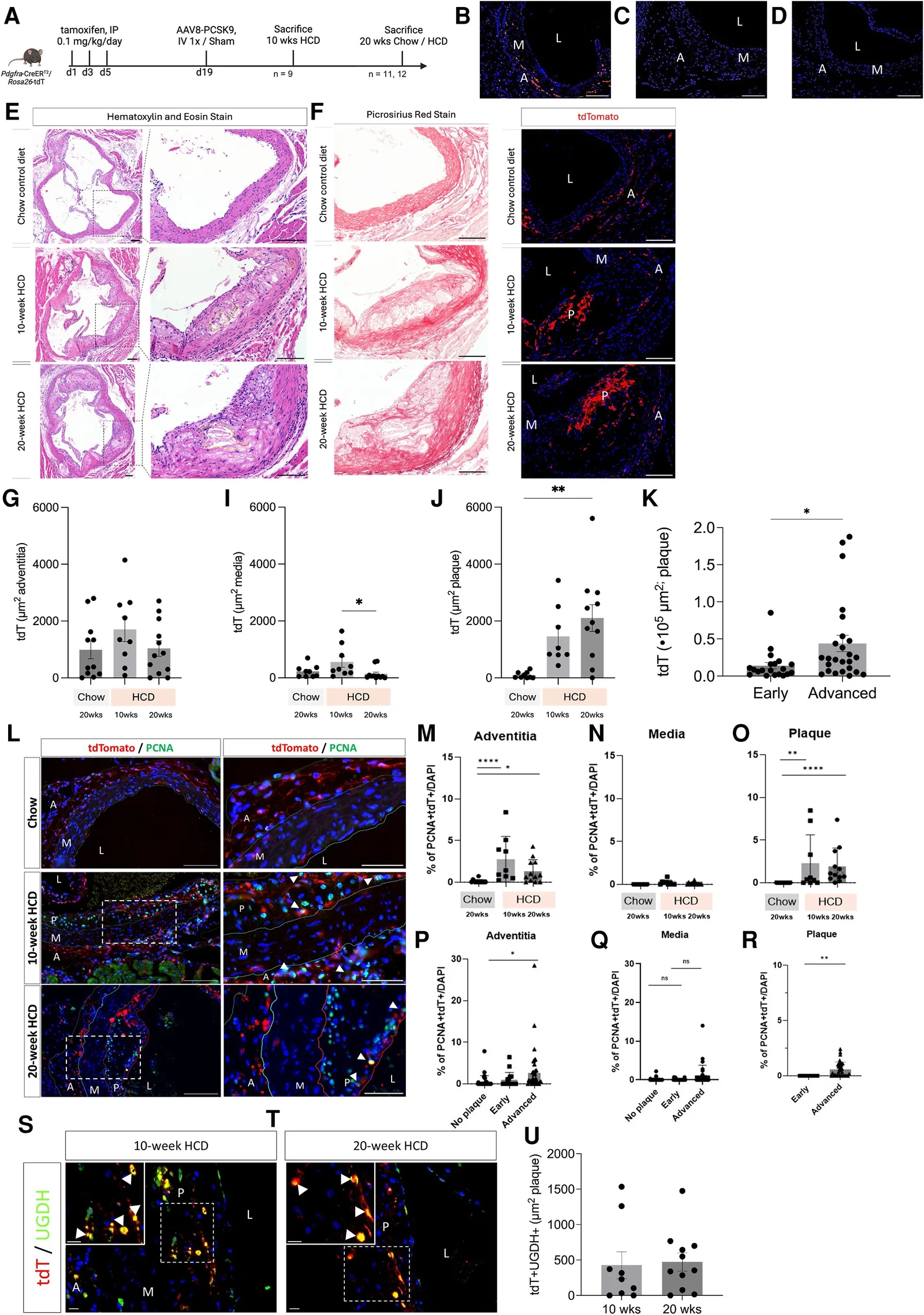
Pdgfra lineage-traced cells populate the atherosclerotic lesion. (*A*) Cartoon depicting the workflow of *in vivo* studies. (*B*) Representative images of immunohistochemical staining depicting tdTomato (red) localization in the aortic root of *Pdgfra*-Cre^ERT2^/Rosa26-tdT mice injected with Tamoxifen (left panel), or *C*. vehicle and stained with anti-tdT antibody. (*D*) Unstained negative control staining on mice injected with Tamoxifen. (*E*) Representative images of haematoxylin and Eosin staining, *F*. Picrosirius Red staining, and *G*. tdTomato staining (red) of the aortic root of mice on a 20-week standard diet, or 10- and 20-week HCD. (*H*) tdT positive pixels in tunica adventitia, *I*. tunica media, and *J*. plaque (one-way ANOVA with Tukey’s multiple comparison test, *n* = 9–12). (*K*) Area of tdT detection in individual atherosclerotic plaques of the aortic root classified as early or advanced (each dot represents one aortic valve from the aortic root, unpaired two-tailed *T*-test, *n* = 9–12). (*L*) Representative images of PCNA (green) co-localization with tdTomato (red) localization in the aortic root of *Pdgfra*-Cre^ERT2^/Rosa26-tdT mice on chow for 20 weeks, or on HCD for 10 and 20 weeks. (*M*) Quantifications of PCNA-tdT co-localization in adventitia, *N*. media, and *O*. plaque of aortic root of *Pdgfra*-Cre^ERT2^/Rosa26-tdT mice on chow for 20 weeks, or on HCD for 10 and 20 weeks (*n* = 9–12). Statistical significance was determined using a two-tailed Mann–Whitney *U* test. *P–R*. Quantification of PCNA ^+^ tdTomato^+^ (PCNA ^+^ tdT^+^) cells in the adventitia, media, and plaque regions of the aortic root in *Pdgfra*-Cre^ERT2^; Rosa26-tdTomato mice. Lesions were stratified as no plaque, early lesions (intimal thickening/progressive intimal thickening; IT/PIT), and advanced lesions (advanced PIT/fibrocalcific atheroma; advPIT/FCA). Data are presented as percentage of PCNA ^+^ tdT^+^ cells normalized to total DAPI^+^ nuclei within each respective compartment (each dot represents one aortic valve from the aortic root, *n* = 9–12), Statistical significance was determined using a two-tailed Mann–Whitney *U* test. (*S*) Representative spectral microscopy images of immunohistochemical staining showing co-localization of tdTomato staining (red) and UGDH (green) at the aortic root of mice on 10-week, or T. 20-week of HCD. (*U*) Quantification of UGDH-tdT co-localization in plaques. Co-localization is highlighted with white arrowheads (unpaired two-tailed *T*-test, *n* = 9, 11). Scale bars 10 µm. L; lumen, M; media, A; adventitia, P; plaque. *P < 0.05, **P < 0.01 and ***P < 0.001.

In addition, we confirmed the *Pdgfra* origin of progenitor fibroblasts within murine plaques by investigating co-localization of tdT expression with UGDH, a subset marker for progenitor fibroblasts. We found UGDH positive nuclei engulfed by cytoplasmic tdT signal and/or cytoplasmic co-stain of both in plaques of 10- and 20-week HCD mice (*Figure 4S–U*).

In summary, *Pdgfra* Lin^+^ plaque cells display a predominant but not exclusive adventitial origin, populate the murine atherosclerotic plaque and expand with plaque progression.

### 3.5 *Pdgfra* Lin^+^ cells predominantly show fibroblast identity, while a minority express SMC markers, and contribute to *Acta2^+^* cap cells

As the expression pattern in healthy mice and the *Myh11* lineage data of the meta-analysis suggests a dual origin of *Pdgfra* lineage-traced, plaque-resident cells, we analysed the protein co-expression of established SMC markers, MYH11, transgelin (TAGLN), and of αSMA (ACTA2) in tdT^+^  *Pdgfra*-lineage-traced cells. We evaluated the overlap of staining and cytoplasmic tdT expression within SMC membranous co-stain and confirmed that 0.4% of tdT^+^ cells in the plaque after 20-week HCD feeding co-express MYH11, 9% co-express SM22α, and 3% αSMA (*Figure 5A–G*), which did not change with plaque progression (*Figure 5A–G*). Notably, MYH11 expression was low as expected, and likely underestimated co-localization with tdT compared to SM22α and αSMA.

**Figure 5:**
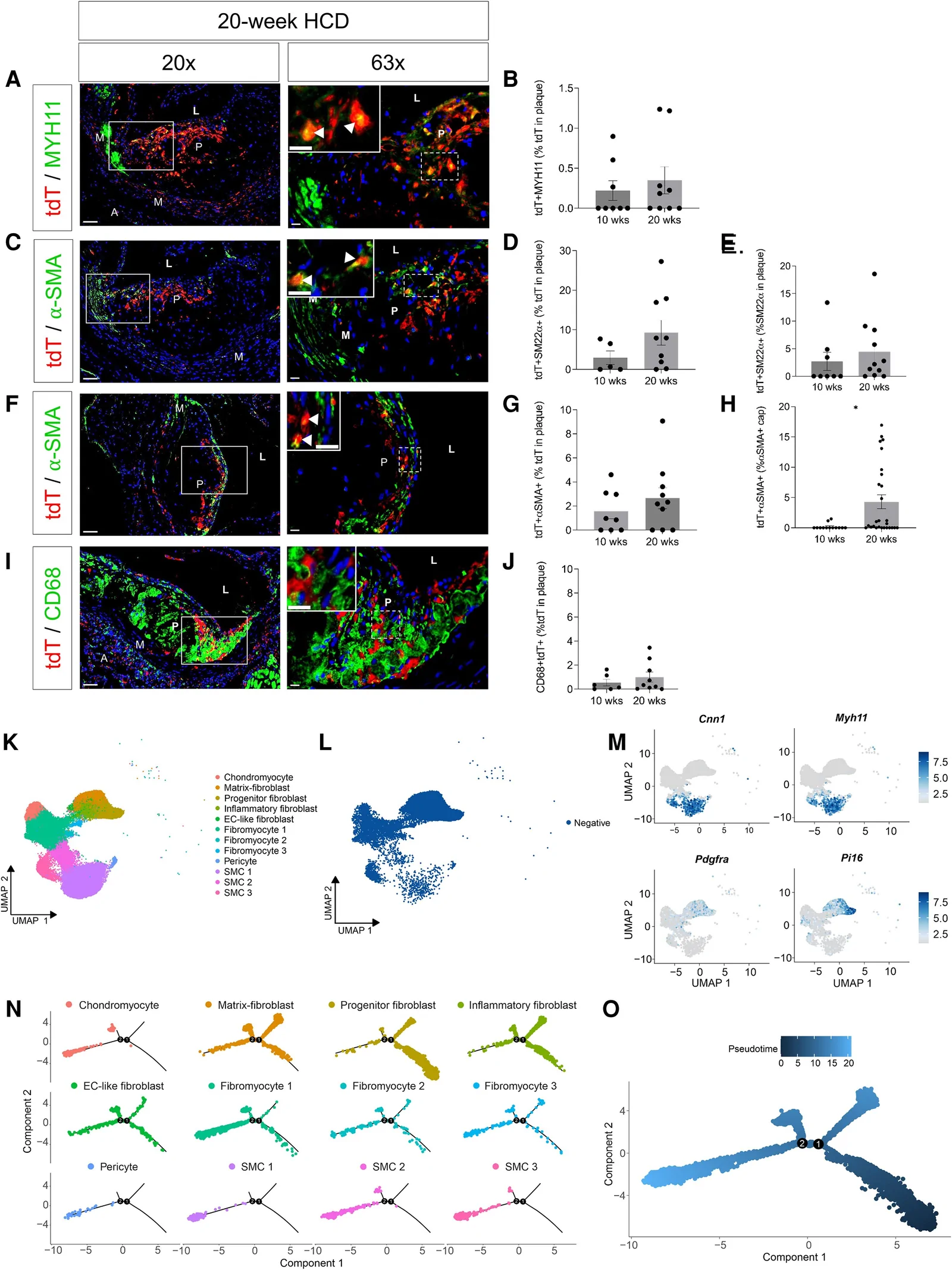
A minority of *pdgfra* lin^+^ cells adopt an SMC-like phenotype in plaque and generate α-SMA^+^ cap cells. *A–J*. Representative spectral microscopy images of immunohistochemical staining and quantifications showing co-localization at the aortic root of tdTomato (red) and cell type markers (green) and quantification of co-localization of marker with tdT area in plaques of mice fed a high cholesterol diet for 10 or 20 weeks in per cent (*n* = 5–11 for all panels, statistical significance was determined using a two-tailed Mann–Whitney *U* test). In H, each dot represents one aortic valve from the aortic root). (*A*) tdT (red) and myosin heavy chain 11 (MYH11, green), and B. quantification. (*C*) tdT (red) and SM22α (green) and *D*. quantification in plaque area and E. in plaque cap area. (*F*) tdT (red) and α-SMA^+^ and *G*. quantification in plaque area and *H*. in plaque cap area. (*I*) tdT (red) and Macrosialin (CD68; green) and *J*. quantification. White squares highlight areas imaged at 63x magnification (right). Dotted white squares highlight zoom-in areas (insets). Co-localization is highlighted with white arrowheads. Scale bars 10 µm. L; lumen, M; media, A; adventitia, P; plaque. *P < 0.05 *K*. UMAP embeddings of meta-analysed fibroblasts, fibromyocytes, chondromyocytes, SMC and pericytes. (*L*) UMAP embeddings of meta-analysed *Myh11*-lineage-negative fibroblasts, fibromyocytes, chondromyocytes, SMC and pericytes. (*M*) Feature Plots showing *Cnn1, Myh11, Pdgfra* and *Pi16* expression within meta-analysed *Myh11*-lineage-negative fibroblasts, fibromyocytes, chondromyocytes, SMC and pericytes. *N–O*. Monocle2 trajectory analysis displayed by the indicated cell clusters (meta-analysed *Myh11*-lineage-negative fibroblasts, fibromyocytes, chondromyocytes, SMC and pericytes) and pseudotime. Fibroblasts had a trend developing into SMC.

We also observed tdT^+^ cells in the cap area. A prior report suggested that 20–40% of the αSMA^+^ cells in the cap of the plaque were not derived from *Myh11* lineage, but also from endothelial or myeloid origin.^63^ Here, we show that 4% of αSMA^+^ cap area is derived from the *Pdgfra* lineage after 20 weeks of HCD. This represented a significant increase from 0.2% after 10 weeks of HCD (*Figure 5H*). These data confirm a dual origin of *Pdgfra*-lineage-traced cells.

However, fibroblasts are very plastic cells, known as a source of inducible pluripotent stem cells, and the atherosclerotic plaque is a conducive environment for cell type (trans-)differentiation. Histology of the *Pdgfra* lineage tracing mouse is unable to unequivocally resolve a SMC fate of plaque-resident fibroblasts due to the low expression of *Pdgfra* Lin^+^/tdTomato in medial SMCs. Thus, a SMC-like fate of plaque fibroblasts was investigated further using single-cell sequencing and lineage tracing. In the meta-analysis presented in *Figure 1*, 5.8% of the *Myh11* Lin^−^ cells were annotated as SMCs. These *Myh11* Lin^−^ cells expressed established SMC markers *Cnn1* and *Myh11*, but not the fibroblast genes *Pdgfra* and *Pi16* (*Figure 5K–M*). The absence of the *Myh11* lineage marker suggested that other cell types may also contribute to the development of plaque SMCs. Indeed, pseudotime analysis performed on *Myh11* Lin^−^ cells shows progenitor fibroblasts are abundant at the start and give rise to two developmental trajectories: one leading to fibroblast subsets and few fibromyocytes, and the other progressing towards SMCs and chondromyocytes (*Figure 5N* and *O*; Supplementary material online, *Figure S7A*). In addition, cell development potential prediction supported a multipotent progenitor state for the progenitor fibroblasts and a differentiated state for *Myh11* Lin^−^ SMCs (see Supplementary material online, *Figure S7B* and *C*). Thus, pseudotime analysis predicts the differentiation of non-*Myh11*-derived SMCs from plaque progenitor fibroblasts. Interestingly, the transcriptome of non-*Myh11*-derived SMCs, fibromyocytes, and chondromyocytes showed heightened expression of inflammatory and fibrotic pathways, reflecting a more synthetic phenotype, compared to *Myh11*-derived cells (see Supplementary material online, *Figures S8* and *S9*).

Additionally, we performed immunohistochemical staining to evaluate the co-localization of tdT and CD68. On average, 1% of tdT^+^ cells showed CD68 expression (*Figure 5I* and *J*), in line with very low level and frequency of macrophage marker expression in plaque fibroblasts (see Supplementary material online, *Figure S7D*). This suggests that relatively few fibroblasts become macrophage-like, despite the detection of an inflammatory fibroblast subset.

These data suggest that *Pdgfra* Lin^+^ cells predominantly maintain fibroblast identity, while less than 10% of *Pdgfra* Lin^+^ cells in the plaque express SMC or macrophage markers.

### 3.6 *Pdgfra* Lin^+^ PECAM1^+^ luminal ECs are distinct from fibroblasts acquiring an EC-like transcriptome

In addition to a SMC or macrophage-like transition, we further dissected the identity, origin and fate of cells with mixed endothelial and fibroblast identity: tdT^+^PECAM1^+^ ECs lining the lumen, identified using the Pdgfra Lin mice, and PECAM1^+^ EC-like fibroblasts observed in the meta-analysis. First, we studied the quantity and location of PECAM1 protein expression in tdT^+^  *Pdgfra* Lin^+^ cells in the atherosclerotic plaques of the aortic root (*Figure 6A–C*). Multispectral analysis showed that 0.5–0.7% of PECAM1^+^ cells in the plaques co-express tdT after 10 and 20 weeks of HCD, respectively (*Figure 6C*). PECAM1^+^/tdT^+^ double positive cells were located on the aorta lumen, in the cap region and deeper in the plaque, and increased with atherogenesis (*Figure 6A* and *C*). We complemented these data with confocal microscopy and could visualize cytoplasmic tdT stain within PECAM1 positive cells in plaques at the aortic root and in the brachiocephalic artery (*Figure 6D*).

**Figure 6:**
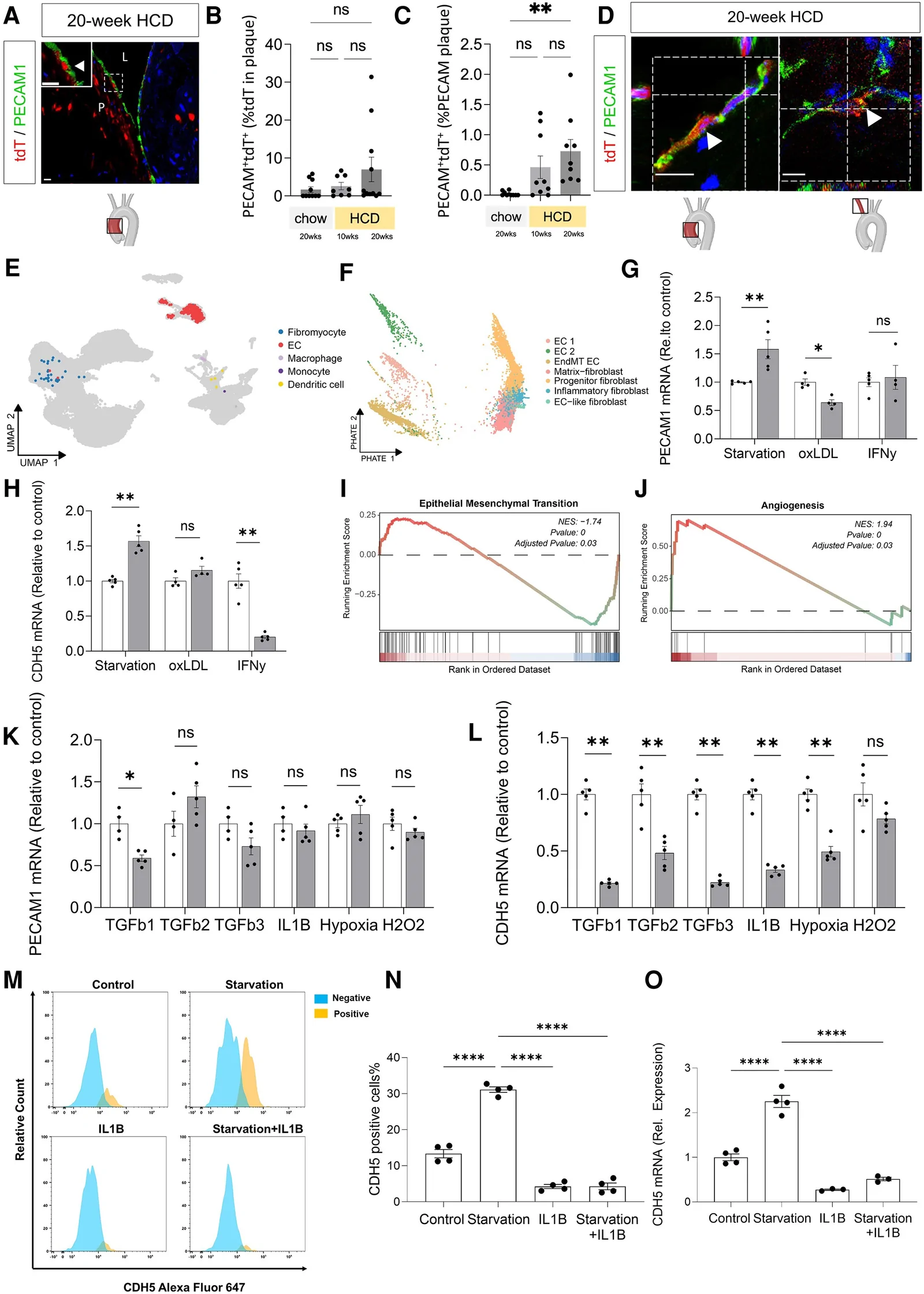
*Pdgfra* lin^+^ plaque fibroblasts have an endothelial cell (EC)-like fate. (*A*) Representative spectral microscopy image of immunohistochemical stains with tdTomato (red), and endothelial marker PECAM1 (green) after 20-week high cholesterol diet (HCD) at the aortic roots of *Pdgfra*-Cre^ERT2^/*Rosa26*-tdT mice injected with tamoxifen. Co-localization is highlighted with white arrowheads. Scale bars 10 µm. (*B*) Quantification of PECAM1 ^+^ tdT^+^ areas as percentage of plaque area, *C*. or PECAM1 area in the plaque *Pdgfra*-Cre^ERT2^/*Rosa26*-tdT mice after 20 weeks of standard diet, 10 or 20 weeks of HCD (*n* = 8–10, one-way ANOVA with Tukey’s multiple comparison test). (*D*) Representative confocal microscopy images of immunofluorescent stains with tdTomato (red), and PECAM1 (green) in plaques of the aortic root (left) or the brachiocephalic artery (right) after 20-week HCD feeding of *Pdgfra*-Cre^ERT2^/*Rosa26*-tdT mice injected with tamoxifen (*n* = 9–10). Scale bars 50 µm for overview, 10 µm for insets. Co-localization is highlighted with white arrowhead. (*E*) UMAP embeddings of meta data showing *Cdh5* lineage-traced cells within each cell cluster. *Cdh5*-lineage-positive cells are coloured by cell types. (*F*) PHATE embeddings of *Myh11 Lin*^−^ fibroblast subsets and endothelial cells. *G–H*. Expression of endothelial markers PECAM1 and CDH5 mRNA in human aortic fibroblasts after starvation (0% FBS), oxidized LDL (25 µg/ml) or IFNy (0.2 µg/ml) stimulation for 24 h (*n* = 4–5). (*I*) GSEA analysis of the meta-analysed EC-like fibroblasts against the HALLMARK gene set in MSigDB for epithelial-mesenchymal transition (EMT) and J. angiogenesis pathways. NES, normalized enrichment score. *K–L*. Expression (fold-change relative to control) of endothelial markers PECAM1 and CDH5 mRNA measured by qPCR in human aortic fibroblasts incubated with TGFb1 (50 ng/ml), TGFb2 (50 ng/ml), TGFb3 (50 ng/ml), IL1B (0.2 µg/ml), Hypoxia (1% oxygen) or H2O2 (25 uM) for 24–72 h or treated with vehicle (*n* = 4–5). Write bar and grey bar represents the control group and the stimulation group, respectively. (*M*) Representative histograms and *N*. quantification of endothelial marker CDH5 analysed by flow cytometry in human aortic fibroblasts subjected to starvation (0% FBS) or/and IL1B (0.2 µg/ml) (*n* = 4). (*O*) Expression of CDH5 mRNA measured by qPCR in human aortic fibroblasts subjected to starvation (0% FBS) or/and IL1B (0.2 µg/ml) (*n* = 4). *M–O*. Representative experiment of three human donors, biological replicates shown in Supplementary material online, *Figure S12*. L; lumen, P; plaque. Statistical analysis was performed using a two-tailed Mann–Whitney test, comparing stimulation group with its corresponding control (G-H and K-L), or one-way ANOVA followed by Tukey’s multiple comparisons test (N–O). For N–O, normality was assessed using the Shapiro–Wilk test. All results show mean ± SEM. *P < 0.05, ** *P* < 0.01, ***P < 0.001, ****P < 0.0001.

While we cannot exclude migration of luminal Pecam1 ^+^ tdT^+^ cells into the cap region, on the transcriptome level, we did not observe -lineage-positive (*Cdh5* Lin^+^) cells in the fibroblast cluster (*Figure 6E, F*). Rather, an EC subset showing a gene signature of EndoMT established using *Cdh5*-lineage tracing^64^ was included in the endothelial *Cdh5* Lin^+^ cluster (see Supplementary material online, *Figure S10A* and *B*). This subset exhibited increased enrichment of the epithelial-to-mesenchymal transition (EMT) pathway, including genes similar to EndoMT, as well as the TGF-β and WNT signaling pathways—both well-known promoters of EndoMT,^65^ compared with other EC subsets (see Supplementary material online, *Figure S10C* and *D*). Furthermore, differentiation patterns in the transcriptional data were further investigated using the algorithm PHATE, confirming that murine plaque ECs were transcriptionally very divergent from EC-like fibroblasts (*Figure 6F*). This suggests that the EC-like fibroblast subset in the *Cdh5*-lineage-negative (*Cdh5* Lin^−^) fibroblast cluster is distinct from tdT^+^ luminal ECs and EndMT^+^  *Cdh5* Lin ^+^ ECs. As aforementioned, pseudotime analysis of the fibroblast subsets already suggested that EC-like fibroblasts derive from progenitor fibroblasts. The lineage tracing study and transcriptional analysis thus suggest it is likely that EC-like fibroblasts derive from progenitor, *Cdh5* Lin^−^ fibroblasts and maintain fibroblast identity, while *Pdgfra* Lin^+^ luminal EC may also contribute to tdT^+^ plaque-resident cells.

### 3.7 Human aorta fibroblasts become EC-like *in vitro* following starvation

We further investigated what could trigger the development of EC-like fibroblasts. Cell starvation *in vitro,* likely triggering autophagy, has been shown to induce the differentiation of cardiac fibroblasts into ECs, a process termed mesenchymal-to-endothelial transition (MET).^66^ Starvation for 24 h in serum-free medium increased expression of established EC markers *PECAM1* and *CDH5* in primary human aortic adventitial fibroblasts (*Figure 6G* and *H*), while levels remained lower than those of primary EC (see Supplementary material online, *Figure S1*). In contrast, oxidized low-density lipoprotein (oxLDL) and interferon-gamma (IFNγ), which mimic the dyslipidemic and inflammatory plaque milieu, respectively, reduced the expression of *PECAM1* or *CDH5* (*Figure 6G* and *H*).

Pathway analysis of the gene signature in EC-like fibroblasts showed decreased enrichment of epithelial to mesenchymal transition, including genes similar to EndoMT, and upregulation of genes related to angiogenesis in EC-like fibroblasts compared to other fibroblast subsets (*Figure 6I* and *J*). Following synchronization of human fibroblasts by quiescence in 0.5% serum, cells were exposed to triggers known to induce EndoMT in atherosclerosis, such as transforming growth factor beta (TGFβ), interleukin 1 beta (IL1B), hypoxia, and hydrogen peroxide (H_2_O_2_).^8^ Indeed, the majority of these EndoMT-promoting stimuli suppressed expression of EC marker (*Figure 6K* and *L*). *Pdgfra* expression was also frequently suppressed (see Supplementary material online, *Figure S11A*). Importantly, IL1B stimulation reversed *CDH5* gene and protein expression induced by starvation in human primary aorta fibroblasts of multiple donors (*Figure 6M–O*; Supplementary material online, *Figures S11B* and *S12*). Notably, cell viability was unchanged, excluding artefacts due to cell death upon starvation or/and IL1B stimulation (see Supplementary material online, *Figure S11C*). Thus, conversion of human fibroblast into an EC-like fate is recapitulated *in vitro* by starvation and can be reversed by stimulation with IL1B.

### 3.8 Four murine plaque fibroblast subsets are conserved to human, four human subsets are unique for human plaques, and one murine subset is murine-specific

While we validated the distinct identity of EC-like fibroblasts in mice, it is imperative from a translational perspective to see if this subset and others are also present in human atherosclerotic plaques. Therefore, we integrated scRNA-seq data from human coronary arteries without macroscopic signs of atherosclerosis,^27^ with data from human coronary arteries with atherosclerosis,^3^ both derived from subjects with heart failure undergoing a heart transplantation (*Figure 7A*; Supplementary material online, *Figure S13A* and *B*). Cells were annotated using cell type markers of a published human vascular meta-analysis (see Supplementary material online, *Figure S13C*).^67^ Human plaque fibroblasts were subsetted (20 867 cells) (*Figure 7B*; Supplementary material online, *Figures S13D* and*S14A*) and integrated with murine plaque fibroblasts, confirming conservation between species (*Figure 7C* and *D*). The integrated analysis identified seven transcriptionally distinct fibroblast subsets across both species (*Figure 7C*), harbouring 8 human subsets and 4 murine subsets (*Figure 7D*; supplementary material online, *Figure S13E*). Human-derived fibroblast subsets co-clustered with murine-derived progenitor, matrix, inflammatory fibroblasts, and EC-like fibroblasts (*Figure 7D*). Immunohistochemistry confirmed detection of progenitor fibroblast marker PI16 in plaques (*Figure 7F*). Co-localization of fibroblast markers PI16 or PDGFRA with endothelial marker PECAM1 was observed in spindle-like cells in the plaque body, as well as in plaque microvasculature. This confirmed the presence of cells with a mixed fibroblast and endothelial state, i.e., EC-like fibroblasts and/or ECs undergoing EndMT, in human atherosclerosis (*Figure 7F*; Supplementary material online, *Figure S13F* and *G*). Interestingly, human EC-like fibroblasts showed high expression of a large number of established EC genes compared to other subsets and to murine EC-like subsets (*Figure 7E* vs. *Figure 1E*), and downregulation of the epithelial to mesenchymal transition pathway (see Supplementary material online, *Figure S13H*). Notably, pathways enriched in human EC-like fibroblasts were more pronouncedly related to inflammation than were murine EC-like fibroblasts (see Supplementary material online, *Figure S14C*; Supplementary material online, *Table S7*).

**Figure 7:**
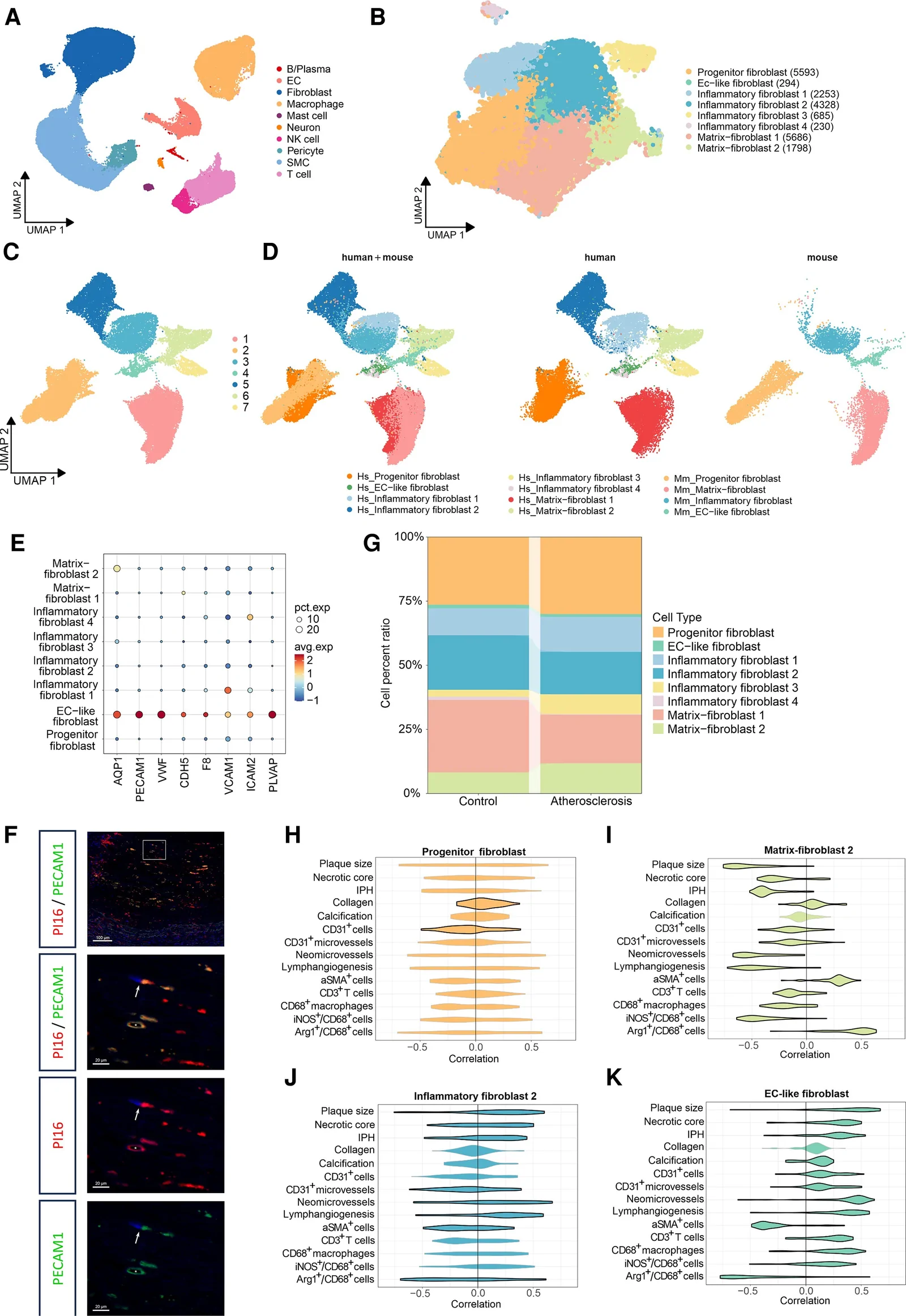
Murine fibroblasts are conserved in human plaques and gene signatures correlate to plaque traits. (*A*) UMAP embeddings of 72 360 single cells from 13 sequencing libraries across 2 studies are colour-coded by cell types. (*B*) UMAP visualization of human fibroblast subsets, derived from fibroblasts in A, colour-coded by subset; numbers in parentheses denote cell counts. (*C*) UMAP embeddings of the cross-species integration of mouse and human fibroblast subsets. (*D*) UMAP embeddings showing species of origin jointly or separately and colour-coded by species subset. (*E*) Dot plot of established marker genes of endothelial cells. (*F*) Representative images of an overview (top) and detailed zoom of human atherosclerotic plaques derived from carotid endarterectomy of symptomatic patients co-stained with fibroblast marker PI16 (red) and endothelial marker PECAM1 (green). Box in overview in top image indicates location of zoom, Co-expression (yellow) is apparent in spindle-like cell (arrow) and in cells lining the plaque microvasculature (*). (*G*) Proportions of fibroblast subsets in control and atherosclerosis samples. *H–K*. Violin plots depicting correlations of the top 50 differentially expressed genes for individual fibroblast subsets with plaque traits in 46 human carotid plaque segments of 23 patients. Significant violin plots (*P* < 0.05) were denoted with a black border. Significance was assessed by positive and negative correlations and the imbalance thereof, which was defined as the sum of positive correlations minus the sum of absolute values of negative correlations. Furthermore, correlation skewness was compared between differentially expressed genes and a random gene set containing a similar number of genes. The permutation test was performed 100 000 times and the *P*-value was the frequency of the random gene sets that have higher correlation skewness than the differentially expressed gene sets.

One further human subset also overlapped partially with murine inflammatory and was named Hs_inflammatory fibroblasts 2 accordingly (*Figure 7D*). Three additional human subsets were distinct from murine subsets, and were named Hs_Matrix fibroblast 2, Hs_inflammatory fibroblasts 3 and 4 respectively based on differential gene and pathway analysis (*Figure 7D*; Supplementary material online, *Figure S14A*, *C*; Supplementary material online, Table  *S7*). Notably, adventitia-restricted murine *Col5a3^+^* fibroblasts were not identified in the integrated human and mouse dataset, even though these human coronary arteries from heart donor transplants should include the adventitia (see Supplementary material online, *Figure S14B*). Adventitial *Col5a3^+^* fibroblasts may be murine-specific. Thus, murine fibroblasts, including EC-like fibroblasts, were indeed transcriptionally conserved between species, while four potential species-specific subsets were detected.

To analyse the influence of human atherosclerosis on fibroblast subset specification, we then characterized the cell proportions of fibroblast subsets in control and atherosclerotic arteries. Human progenitor, EC-like fibroblasts, inflammatory fibroblast 1 and 3, and matrix fibroblast 2 showed a relative enrichment in samples with atherosclerosis vs. controls, while proportions of matrix fibroblast 1, inflammatory fibroblast 2 and 4 declined (*Figure 7G*). To provide a further translational perspective, the individual associations of all genes differentially expressed by human fibroblast subsets with human carotid plaque traits quantified in 43 plaques were determined in the Maastricht human plaque cohort (AtheroMAAS).^26^ This revealed that gene signatures of progenitor fibroblasts and matrix fibroblast 2 were positively correlated with plaque traits marking plaque stability, i.e. plaque collagen content, anti-inflammatory macrophages, and ACTA2^+^ cells (*Figure 7H* and *I*). The matrix fibroblast 1 and 2 subsets were also negatively correlated with plaque instability traits, like plaque size, necrosis and IPH (*Figure 7I*; Supplementary material online, *Figure S14D*). In contrast, gene signatures of four human plaque fibroblasts, i.e. EC-like fibroblasts and inflammatory fibroblasts 1, 2 and 4, were mostly positively correlated with plaque traits that signify plaque instability, such as plaque size, IPH, necrotic core content, and inflammatory cell densities (*Figure 7J* and *K*; Supplementary material online, *Figure S14E* and *F*). Inflammatory fibroblasts 3 did not correlate with any plaque traits (see Supplementary material online, *Figure S14G*).

In conclusion, human fibroblast subsets recapitulated murine progenitor fibroblasts, matrix fibroblasts, inflammatory fibroblasts, and EC-like fibroblasts (*Graphical abstract*), and were associated differentially with atherosclerotic plaque traits. Human plaques show four human-specific subsets. Human plaque fibroblasts overall showed a more pronounced inflammatory profile and associations with plaque instability traits.

## 4. Discussion

In the current study, we assessed atherosclerotic plaque fibroblast identity, origin and fate using analysis of scRNA-seq data of 54 RNAseq libraries datasets derived from adventitia or plaques, and exploiting *in vivo* lineage tracing studies with *MYH11*, *CDH5*, and *PDGFRA* reporter mice (*Graphical abstract*). We found that human and murine plaque fibroblasts are marked by *Pdgfra*, *Pi16*, *Cygb*, and *Serpinf1* mRNA expression. *PDGFRA* lineage tracing of adventitial fibroblasts, induced before atherogenesis, revealed that plaque fibroblasts were predominantly of adventitial origin with a smaller contribution of medial MYH11^+^ cells. Beyond fibroblast origin, our integrated mouse–human analysis highlights conserved fibroblast heterogeneity as a disease-relevant feature of atherosclerosis. We identified a fibroblast progenitor subset that differentiates into matrix fibroblasts, inflammatory fibroblasts, and EC-like fibroblasts in mice, which were all conserved in human plaques. In addition to these conserved subsets, human plaques contained additional fibroblast subsets, likely reflecting species differences, co-morbidities, and the more complex microenvironment of human atherosclerosis. *Cdh5* Lin^−^, EC-like fibroblasts arising via MET were validated by lineage tracing and *in vitro* studies, and were distinct from EndMT-ECs. Progenitor and matrix fibroblast signatures were associated with plaque stability traits, whereas EC-like fibroblasts and inflammatory fibroblast subsets were linked to human plaque instability traits. These associations suggest that fibroblast state transitions may represent potential therapeutic entry points to modulate plaque phenotype and vulnerability. *Pdgfra* Lin*^+^* fibroblasts predominantly maintained fibroblast identity, and only a small proportion expressed SMC, EC or macrophage markers, or transitioned into *Myh11* Lin^−^ SMC, fibromyocytes, and chondromyocytes. Together, this study offers the field gene markers and tools to study the plaque fibroblast population as a whole, its subsets and fibroblast-derived cells as a new niche within the field of atherosclerosis.

A thorough and detailed identification of transcriptionally distinct fibroblast clusters was missing until now, although fibroblast heterogeneity was apparent from murine and human scRNA-seq data.^2,3,6,7^ Recently, we identified and validated the expression of *Pdgfra* as an identity marker of adventitial fibroblasts in healthy murine and human arteries.^7^ Here we elucidate the origin of plaque fibroblasts, which are predominantly derived from *Pdgfra* Lin^+^, *Myh11* Lin^−^ adventitial cells, with a smaller contribution (6%) of *Myh11* Lin^+^ SMCs. The transient increase in medial *Pdgfra* Lin^+^ content, in the absence of detectable medial proliferation is further in support of this adventitial contribution. This observation suggests that *Pdgfra* Lin^+^ fibroblast-lineage cells accumulate in plaques through migration into the lesion and subsequent local proliferation. This predominant adventitial contribution concurs with recent non-lineage tracing studies using CYTOF and vessel wall layer-specific sequencing to suggest adventitial origin,^68,69^ and with prior observations of SMC to fibromyocyte differentiation.^3^ In carotid artery ligation, a model mimicking restenosis, medial *Pdgfra* Lin^+^ Myh11^+^ cells were more frequently observed to invade the neo-intima, albeit a different Pdgfra-Cre transgene was employed.^70^ This underscores that fibroblast plasticity is contextual rather than invariable. Consequently, functional or gene-silencing studies using *Pdgfra*-Cre^ERT2^ in atherosclerosis should account for the dual *Pdgfra* Lin^+^ population, including predominantly adventitial fibroblast-lineage cells and a minor medial SMC-derived cells.

Before the advent of scRNA-seq and reliable lineage reporter models, activated fibroblasts expressing *Acta2* were termed myofibroblasts.^12^ However, in the *Pdgfra*-lineage tracing model, only 9% of tdT^+^ plaque cells expressed ACTA2 at the protein level in mice, indicating that most Pdgfra-lineage plaque cells did not acquire a classical ACTA2^+^ myofibroblast phenotype. In the scRNA-seq meta-analysis, *Acta2* mRNA was detected in 87% of *Myh11* Lin^+^ fibroblasts, compared with 33% of *Myh11* Lin^−^ fibroblasts, and *Myh11* Lin^+^ fibroblasts showed higher Acta2 expression levels, indicating that ACTA2^+^ fibroblast-like states can include SMC-lineage-derived cells. In parallel, in human atherosclerosis, *ACTA2* was expressed by 30–60% of plaque fibroblasts, depending on the subset. As ACTA2 is expressed by both SMCs and fibroblasts,^7^ this suggests that phenotyping fibroblast subsets based on ACTA2 alone does not allow a conclusion on identity and origin.

Determining the origin further, adventitial *Pdgfra Lin*^+^  *Ugdh^+^* progenitor fibroblasts were identified as the main source for plaque fibroblasts, in line with a report of an adventitial CD26 ^+^ CD55^+^ subset.^69^ Interestingly, the progenitor subset does not fully recapitulate the expression pattern of traditionally termed resident mesenchymal stem cells.^62^  *Ly6a^+^* progenitor cells have been implied in atherosclerosis, vein grafts or neointima formation before.^16,18,21,71^ However, these investigations either included all *Ly6a^+^* cells irrespective of haematopoietic, myeloid or mesenchymal identity, or focused on *Ly6a*/*Myh11* double positive cells, effectively excluding fibroblasts.^16,18,21,71^ Also, controversy exists as not all studies report a significant contribution of *Ly6a^+^* or other multipotent progenitors to SMCs in atherosclerosis.^72,73^ In contrast, compelling evidence supports medial SMCs de-differentiation into a progenitor state marked by *Ly6a* and *Gli1* in homeostasis. Upon vascular injury or atherosclerosis, these cells subsequently transition into myofibroblasts, fibroblasts, or become SMCs yet again.^18,74^ As a small fraction of *Pdgfra* Lin^+^ cells in our study populate the media, and partly express Myh11 or Tagln, this suggests overlap with the *Ly6a*/*Myh11*-double positive cells. Possibly these could be the origin of the *Myh11* Lin*^+^* fibroblasts observed in the murine plaque meta-analysis described here. This is in line with observations of Pdgfra-derived cells, positive for Sca1^+^, differentiating into SMCs in a related, but distinct, normocholesterolemic vascular injury pathologies.^70,71^ This warrants further close comparison between transcriptional profiles across studies, and the creation of nested or intersectional double lineage reporter models. Presumably, a shared embryonic origin can explain the strong resemblance. Nevertheless, the majority of plaque fibroblasts studied in this report did not derive from MYH11 cells, and likely constitute a separate cell identity with a different function. These prior data, together with our lineage tracing, point to the adventitia not merely as a structural wrapper, but as an active, disease-responsive mesenchymal niche with a capacity to contribute cells to the plaque compartment.

In addition to identity and origin, we studied the fate of *Myh11* Lin^−^, and *Pdgfra* Lin^+^ fibroblasts in atherosclerosis, and observed conversion into fibromyocytes, chondromyocytes and finally into SMCs. This is in contrast to prior conclusions based on single-cell sequencing data.^60^ Although the paper described a small amount of *Myh11* Lin^+^ cells in the fibroblast fraction, these cells were considered to be cell doublets.^60^ The expression of SMC markers in our *Pdgfra* Lin^+^ plaque cells likely indicates a dual origin from medial *Myh11 Lin*^+^, as well as adventitial *Myh11 Lin*^−^ adventitial origin. Our observations of *Myh11* Lin^−^ fibroblast-derived SMCs are supported by the reported conversion of fibroblasts into SMCs *in vitro*.^75^

In parallel to fibroblast-SMC transitions, the fate of progenitor fibroblasts was pinpointed to further fibroblast subsets. Interestingly, we observed the expression of EC markers *PECAM1*, *CDH5*, and *VWF* by human and murine EC-like fibroblasts. Although EC-like fibroblasts were less abundant than other fibroblast subsets, their presence was supported by detection across independent mouse and human datasets, high internal homogeneity, comparable QC metrics, and validation in intact murine and human plaque tissue sections. In support of this interpretation, previous studies identifying rare fibroblast populations, including subsets comprising ≤4% of fibroblasts or as few as 59 cells, confirmed their presence in intact tissue sections.^76,77^ A recent independent study also reported plaque fibroblasts with endothelial-marker expression,^78^ further supporting that this phenotype is not specific to our analysis. Together, these findings support the validity of the EC-like fibroblast population and indicate that it is unlikely to reflect technical noise, dissociation artefacts, or unstable clustering.

While these cells with dual markers could originate from ECs via EndMT^8^ or from fibroblasts, *Cdh5* lineage tracing presented here does not support endothelial derivation of EC-like fibroblasts. The *Cdh5* Lin^−^, EC-like fibroblasts may thus result from MET. Starvation-triggered MET was recently uncovered in the heart, contributing to cardiac neovascularization, and ameliorating cardiac dysfunction after myocardial infarction.^66^ The direct conversion of fibroblasts into ECs has previously been reported.^79^ Actually, transition into an EC state is not uncommon in atherosclerosis, as contractile SMCs are also able to transdifferentiate into an EC-like phenotype *in vitro* and *in vivo*.^80,81^ Functionally, EC-like fibroblasts could present a source of luminal ECs to maintain EC coverage on the surface of a rapidly growing plaque. However, this subset could be detrimental to plaque stability if the resulting barrier function would be compromised, or if EC-like fibroblasts increase the display of pro-inflammatory adhesion molecules. Their adverse effect is suggested by their association with plaque instability.

In contrast, the effect of matrix-fibroblasts is likely stabilizing. Indeed, *Pdgfra*-driven tdT^+^ expression in murine plaques correlated with collagen content and necrosis. Our prior work also supports plaque fibrosis resulting from fibroblast-macrophage crosstalk.^59^ Additionally, depletion of fibroblast-like cells marked by proteoglycan 4 (PRG4) leads to the thinning of the plaque cap.^10^ However, PRG4 was not only expressed by fibroblasts, but also by SMCs and senescent cells. Both studies were published before the proper validation of plaque fibroblast identity in this report, thus neither study convincingly confirmed the fibroblast identity of the cells underlying the observed pro-fibrotic phenotype. The function of fibroblasts and different subsets thus requires elucidation by further causality studies beyond the scope of the current manuscript.

Limitations from our study are inherent to all lineage tracing reporter models, such as not being wholly specific or sensitive. We previously identified endogenous PDGFRA expression as a specific adventitial fibroblast marker across healthy vascular beds.^7^ Here, we noted the medial expression of the *Pdgfra*-driven tdT reporter (0.05% of medial area) in healthy arteries, and co-localization of SM22α and TdT in 1% of the medial SM22α area. Thus, clonal expansion of proliferating medial cells with this shared SMC-fibroblast identity could also explain part of the PDGFRA-derived cells in the plaques, in addition to possible adventitial fibroblast migration via the media. However, as proliferating PCNA^+^  *Pdgfra Lin*^+^ cells are hardly present in the media, migration is most likely and in line with prior reports.^50,68,69^ Full confirmation is needed by future studies using a multi-colour fluorescent reporter expression governed by *Pdgfra*-driven Cre recombinase and/or a *Myh11-Pdgfra* nested reporter model.

In parallel, the *Myh11* reporter may not be 100% efficient, so a fraction of *Myh11* Lin^−^ fibroblasts and SMCs can still derive from *Myh11* Lin*^+^* SMCs. Furthermore, in our mouse metadata, *Myh11* Lin^+^ and *Myh11* Lin^−^ cells are found in the same cluster, suggesting transcriptional similarity. However, their functions might be different as suggested by enhanced inflammatory and fibrotic pathway expression in fibroblast-derived SMCs. Furthermore, *Cdh5*-lineage tracing data was based on 600 cells, not allowing a wholly solid conclusion on the absent endothelial origin of all EC-like plaque fibroblasts. Nevertheless, concurrent co-expression of endothelial and fibroblast proteins in human plaque cells, which do not bear lineage tracing, should in the future be interpreted carefully regarding the cell type of origin. In addition, conclusions on human conservation and subset proportion changes are based on 13 human samples, which may not fully represent human heterogeneity. As a further limitation, the pronase/collagenase-based digestion protocol used for the adventitia and plaque + media scRNA-seq analysis was optimized for fibroblast recovery; therefore, quantitative interpretation of medial SMC abundance from this dataset is limited.

In conclusion, adventitial and medial *Pdgfra Lin^+^* form a dual origin of plaque fibroblasts, while 95% of plaque fibroblasts are *Myh11 Lin*^−^. *Pdgfra* Lin^+^ cells largely maintain fibroblast identity, while few cells express SMC, EC or macrophage markers. Murine plaque fibroblasts are present in four subsets with different Gene Ontology and are conserved in human plaques. The functional consequences of the fibroblast subsets and fibroblast-derived SMCs require further study to explore potential new therapeutic options for atherosclerosis.

Translational Perspective High residual risk of cardiovascular events (CVE) with current therapy suggests improved insight into drivers of atherosclerotic plaque stability is required. While plaque fibroblasts could represent a new therapeutic target, their identity needs resolving. Thus, the identity, origin, and fate of plaque fibroblasts were resolved by integrating lineage tracing with cross-species single-cell analyses. Plaque fibroblasts predominantly arise from adventitial *Pdgfra*-lineage progenitor fibroblasts. Murine fibroblast subsets are conserved in human atherosclerosis, and distinct human fibroblast states are associated with plaque stability or instability traits. These findings define plaque fibroblast identity and support fibroblast state transitions as clinically relevant targets for future plaque-stabilizing therapies.

## Supplementary Material

cvag183_Supplementary_Data

## Data Availability

All raw and processed scRNA data produced in this study are available in the GEO database and are publicly available as of the date of the publication under the GEO accession number GSE277853. Code is deposited in Zenodo ( https://doi.org/10.5281/zenodo.18250729) and publicly available as of the date of publication. Any additional information required to reanalyse the data reported in this paper is available from the lead contact upon request.
