# Supplementary material for: Atherosclerotic plaque fibroblasts derive from adventitial and medial Pdgfra-lineage-positive cells and predominantly maintain fibroblast identity
Source: Cardiovasc Res. 2026 Sep 9;122(15):2095–115. doi: 10.1093/cvr/cvag183 (PMC13742058; doi:10.1093/cvr/cvag183)
Supplement: Supplementary file 1 [file cvag183_supplementary_data.zip › Rev2_CVR_Supplementary material_clean.pdf]

Supplementary material for:

Atherosclerotic plaque fibroblasts derive from adventitial and medial *Pdgfra* lineage-positive cells, and predominantly maintain fibroblast identity.

Guanliang Li,[1*] Sebastiaan EJ Asselberghs,[1,2*] Baixue Yu,[1*] Cong Feng,[3] Paul Mendez,[1] Dimitri Kasakovski,[1,4] Pieter Goossens,[1] Tia M Ackermans,[1] Renée JHA Tillie,[1] Marion J Gijbels,[1,5] Lieve Temmerman,[1] He Xu,[1] Marjo MPC Donners,[1] Dlzar A Kheder,[1] Sidrah Maryam,[4] Han Jin,[1,6] Chenyi Xue,[7] Sikander Hayat,[4] Austin C Conklin,[8] Casey E Romanoski,[9] Mauro Giacca,[10-12] Clint L Miller,[13] Muredach P. Reilly,[7] Ming Chen,[3] Rafael Kramann,[4,14] Barend Mees,[2] Andrew H Baker, [15,1] Judith C Sluimer[1,4,15]

Content:

Supplementary Figure legends
Supplementary figures 1-14
Supplementary tables 1-7

Supplementary Figure 1. Human aortic adventitial fibroblasts purity
A. Expression of mRNA from cell type specific marker genes (PDGFRA – aortic fibroblasts, ACTA2 – smooth muscle cells, CDH5/PECAM1 – endothelial cells, CD68 – macrophages) determined by qPCR in human aortic fibroblasts (Hfibroblasts) and respective control cells, human vascular smooth muscle cells (HSMCs), human macrophages (HMac), human microvascular endothelial cell line-1 (HMEC-1s) (n=4). **B.** Expression of mRNA from cell type specific marker genes (PDGFRA – aortic fibroblasts, ACTA2 – smooth muscle cells, CDH5/PECAM1 – endothelial cells, CD68 – macrophages) determined by qPCR in human aortic fibroblasts of two additional donors and respective control cells (n=3-5).

Supplementary Figure 2. QC and cluster trees for data in Figure 1
A. ClusterTree of meta-analysis at different resolutions. **B.** Stacked bar plot showing the distribution of different cell types across 4 studies used in meta-analysis. **C–E.** UMAP embeddings of mouse atherosclerosis meta scRNA data, highlighting *Myh11*-lineage-positive, *Myh11*-lineage-negative and no-reporter cells, respectively. In each panel, highlighted cells are colored by cell type, while all other cells are shown in grey. **F.** Dataset-specific proportions of *Myh11*-lineage-positive (green) and *Myh11*-lineage-negative (red) cells of among fibroblasts in the Pan et al. (*Ldlr*$^{-/-}$) and Wirka et al. (*ApoE*$^{-/-}$) datasets. **G.** Violin plot of *Acta2* mRNA in *Myh11* Lin^{+} and *Myh11* Lin^{-} fibroblasts from the Pan et al. (*Ldlr*$^{-/-}$) and Wirka et al. (*ApoE*$^{-/-}$) datasets. **H.** Proportion of *Cdh5*-lineage positive (red) cells and other (grey) cells of each cell type.

Supplementary Figure 3. QC of fibroblast subsets in meta-analysed scRNA data
A. Quality-control metrics of meta-analysed fibroblast subsets. **B.** ClusterTree of meta-analysed fibroblast subsets at different resolutions. **C.** Boxplots showing ROGUE values for progenitor fibroblasts, inflammatory fibroblasts, matrix fibroblasts, and EC-like fibroblasts in the mouse meta atherosclerosis scRNA dataset.

Supplementary Figure 4. Pathway analysis of fibroblast subsets
A. Heatmap of the distribution of significant HALLMARK gene sets identified by irGSEA using robust rank aggregation algorithm (RRA) across fibroblast subsets. The heatmap illustrates whether certain pathways exhibit statistically significant differences in each fibroblast subset. Squares in blue indicate no statistical difference, while squares in red indicate a statistical difference. * indicates $P < 0.05$, ** $P < 0.01$, ***, $P < 0.001$, and ****, $P < 0.0001$. The clustering tree on the left represents the similarity of expression patterns of different pathways in fibroblast subsets. The bars above represent the different fibroblast subsets. The red bars indicate up-regulated differential gene sets, while blue bars represent down-regulated differential gene sets, respectively. **B.** GSEA analysis of the fibroblast subsets for matrisome-related pathways. Matrisome-related pathways are divided into two groups: 1) core matrisome pathways including collagens, proteoglycans and ECM glycoproteins, and 2) matrisome associated pathways including ECM Affiliated, ECM regulators and secreted factors. Matrisome-related pathways are all upregulated in matrix fibroblasts. NES, normalized enrichment score. **C.** UCell enrichment of gene signature of progenitor fibroblast from the meta-analysis in mouse healthy adventitial fibroblasts. The gene signature consisted of the top 20 differential expression genes within meta-analysis progenitor fibroblasts, compared with other fibroblast subsets. **D.** The cellular potency category of 4 fibroblast

subsets in Pan et al. predicted by CytoTRACE 2. Progenitor fibroblasts exhibited the highest developmental potential, followed by inflammatory fibroblasts. **E.** Dot plot of traditional mesenchymal marker expression within 4 fibroblast subsets.

Supplementary Figure 5. QC data of scRNA from chow-HCD adventitia and plaque

A. Quality-controlled data of all cell types from adventitia and plaque with media of LDLR-Ko mice on chow and HCD. **B.** ClusterTree of scRNA data from adventitia and plaque with media from LDLR-Ko mice on chow and HCD at different resolutions. **C.** Clustertree of fibroblast subsets at different resolutions. **D.** UMAP embedding of 6 fibroblast subsets. **E.** The distribution of fibroblast subsets by *Ro/e* analysis. One cluster was identified as being enriched in a specific layer or disease status if *Ro/e* > 1. **F.** Ucell enrichments of gene signatures of matrix, **G.** Col5a3$^+$, **H.** inflammatory and **I.** EC-like fibroblasts on healthy adventitial fibroblast subsets (Cd55$^+$, Cxcl14$^+$ and Lox$^+$) as indicated by labels. **J.** Stacked bar plot showing the proportion of different fibroblast subsets in *ApoE-/-* (Wirka er al.,) and **K.** *Ldlr-/-* (Pan et al.,) mouse atherosclerosis model.

Supplementary Figure 6. PECAM1-tdTomato co-localization across murine arteries and correlation of tdTomato expression with murine plaque traits
A. PECAM1 (green), or **B**. SM22 (green) co-staining with tdTomato (red) in brachiocephalic artery (BCA), abdominal aorta (AA), left carotid artery (LCA) and femoral artery (FA) of 15-17 week-old Pdgfra-CreERT2/Rosa26-tdT mice injected with Tamoxifen fed standard chow (n=11). **C**. Non-parametric Spearman correlation assessments of tdTomato (tdT) and mean atherosclerotic plaque burden, **D.** necrosis area and **E.** collagen content (n=17-21). **F.** Correlation tdT and mean % CD68, **G.** PECAM1, and **H.** MYH11 in atherosclerotic plaques of Pdgfra-CreERT2/Rosa26-tdT mice (n=17-19, linear regression analyses).

Supplementary Figure 7. Pseudo time expression and cellular potency of murine mesenchymal cells
A. Heatmap of top marker genes in different fibroblasts subsets and the established marker genes of chondromyocytes, fibromyocytes, SMC and pericytes in pseudotime, following the trajectory timeline shown in Figure 5O. **B.** The cellular potency category of the indicated cell clusters (*Myh11*-lineage negative fibroblasts, fibromyocytes, chondromyocytes, SMC and pericytes) from the meta-analysis in Figure 1A was predicted by CytoTRACE 2 in data from Wirka et al, and **C.** using data from Pan et al. Progenitor fibroblasts showed the highest developmental potential and were considered as the starting point of differentiation for pseudo-time analysis. **D.** Classical macrophage marker genes expression of fibroblast subsets and macrophage from mouse meta data.

Supplementary figure 8. GSEA of *Myh11*-negative differentially expressed genes compared with *Myh11*-positive across SMC, fibromyocyte, and chondromyocyte
A-C. GSEA of differentially expressed genes (*Myh11*-negative vs *Myh11*-positive) within SMC (**A**), fibromyocyte (**B**), and chondromyocyte (**C**) using the MSigDB Hallmark gene sets.

Supplementary figure 9. Function of differential expression genes between *Myh11*-positive and –negative within SMC, fibromyocyte and chondromyocyte

A. - H. GO enrichment analysis of differential expression gene set between *Myh11*- positive and negative cells among SMC (**A** and **B**), fibromyocyte (**C** and **D**), chondromyocyte (**E** and **F**) and fibroblast (**G** and **H**).

Supplementary figure 10. Identification of EC in EndMT

A. UMAP embeddings of mouse meta-analysis with level 2 annotation, colored by cell types. **B.** Expression of previously defined EndMT EC markers (Shin et al.) among meta-analysed EC subsets. **C.** GSEA of the meta-analysed EndMT EC against the HALLMARK gene set in MSigDB for epithelial-mesenchymal transition (EMT pathway). **D.** Pathway activity inference of EC subsets using decoupleR.

Supplementary Figure 11. PDGFRA mRNA expression, and flow cytometry gating and live cell percentage of cultured human fibroblasts

A. mRNA expression (fold-change relative to control) of fibroblast markers (PDGFRA) measured by qpcr in human fibroblasts incubated with TGFb1 (50ng/ml), TGFb2 (50ng/ml), TGFb3 (50ng/ml), IL1B (0.2ug/ml), Hypoxia (1% Oxygen), H_2O_2 (25uM), starvation (0% FBS), oxidized LDL (25ug/ml), or IFNγ (0.2ug/ml) for 24-72 hours or treated with vehicle (n=4-5). **B.** Gating strategy: Cells were gated, doublets and dead cells were excluded. Cells were gated for CDH5 expression (n=4). **C.** Percentage of live cells in human fibroblasts subjected to starvation (0% FCS) or/ and IL1B (0.2ug/ml) (n=4). L; lumen, M; media, A; adventitia, P; plaque. Statistical analysis was performed using a two-tailed Mann-Whiteney test, comparing stimulation group with its corresponding control (A). All results shown mean ± SEM. $^*P < 0.05$, $^{**}P < 0.01$, or $^{***}P < 0.001$.

Supplementary Figure 12. Starvation induces an endothelial cell (EC)-like fate in human aortic fibroblasts of different donors

A. Quantification of endothelial marker CDH5 analysed by flow cytometry in human aortic fibroblast of two additional donors subjected to starvation (0 % FBS) or/and IL1B (0.2 ug/ml) (n=2). **B.** Expression of CDH5 mRNA measured by qPCR in human aortic fibroblasts of two additional donors subjected to starvation (0% FBS) or/and IL1B (0.2 ug/ml) (n=4-5). Statistical significance was calculated in between samples using one-way ANOVA and Tukey's multiple comparisons test; Normality was tested using Shapiro-Wilk test; All results show mean ± SEM. $^*P < 0.05$, $^{**}P < 0.01$, $^{***}P < 0.001$, or $^{****}P < 0.0001$.

Supplementary Figure 13. Human atherosclerosis meta-analysis and fibroblast subsets

A. ClusterTree of human meta scRNA data with and without atherosclerosis at different resolutions. **B.** UMAP visualization of cells from control and atherosclerosis samples color-coded by cell types **C.** Dot plot of established marker genes of cell types. Dot diameter represents the percentage of cells in certain cell type expressing corresponding genes**. D.** ClusterTree of human meta-analysed fibroblast subsets at different resolutions. **E.** PCA embeddings of the integration among mouse and human fibroblast subsets. **F.** Representative

images of an overview (top) and detailed zoom of human atherosclerotic plaques derived from carotid endarterectomy of symptomatic patients co-stained with fibroblast marker PDGFRA (red) and endothelial marker PECAM1 (green). Box in overview in top image indicates location of zoom, Co-expression is apparent in spindle-like cell (arrow) and in cells lining the plaque microvasculature (*), and **G.** EC lining the lumen. **H.** GSEA analysis of EC-like fibroblast against the HALLMARK gene set in MSigDB for epithelial-mesenchymal transition (EMT) pathway. NES, normalised enrichment score.

Supplementary Figure 14. Markers, gene signature enrichment, functional analysis of human fibroblast subsets and their correlations with plaque traits

A. Dot plot of markers of human fibroblast subsets. **B.** UCell enrichment of top 50 differential expression genes within mouse Col5a3^{+} fibroblast subsets compared with other mouse fibroblast subsets on human fibroblast subsets. **C.** Key functions of human fibroblast subsets based on GO enrichment analysis**. D-G.** Violin plots depicting correlations of the top 50 differentially expressed genes of each fibroblast subset with plaque traits in 43 human carotid plaque segments. Significant violin plots ($p < 0.05$) were denoted with a black border. Significance was assessed by positive and negative correlations and the imbalance thereof, which was defined as the sum of positive correlations minus the sum of absolute values of negative correlations. Furthermore, correlation skewness was compared between differentially expressed genes and a random gene set containing a similar number of genes. The permutation test was performed 100,000 times and the p-value was the frequency of the random gene sets that have higher correlation skewness than the differentially expressed gene sets.

Supplementary Table 1: Sample description murine meta-analysis

Reference (1st author)	PubmedID	GEO	Libraries	Artery	Artery layers	Mouse model	Diet type	Diet duration (weeks)
Pan	32962412	GSE155513	4 SMC Zsgreen^{+}<br>4 Non-SMC Zsgreen^{-}	AA, BA, TA	Plaque, media, adventitia ND	Ldlr$^{-/-}$ Myh11-Cre/ZsGreen	21% fat, 0.2% chol*	0, 8, 16, 26
Pan	32962412	GSE155513	3 SMC Zsgreen^{+}<br>3 Non-SMC Zsgreen^{-}	AA, BA, TA	Plaque, media, adventitia ND	ApoE$^{-/-}$ Myh11-Cre/ZsGreen	21% fat, 0.2% chol*	8, 16, 22
Alencar	32674599	GSE150644	3 SMC eYFP^{+}<br>5 eYFP^{+} and eYFP$^{-\#}$	BA	Only plaque, no media/adventitia	ApoE$^{-/-}$Myh11-Cre/eYFP	21% total fat, 0.2% chol*	18
Alencar	32674599	GSE150644	1 eYFP^{+} and eYFP$^{-\#}$	Aorta	Media, adventitia ND	ApoE$^{-/-}$Myh11-Cre/eYFP	Standard diet	8
Alencar	32674599	GSE150644	1 EC eYFP^{+}<br>1 eYFP^{+} and eYFP$^{-\#}$	BA	Only plaque, no media/adventitia	ApoE$^{-/-}$ Cdh5 Cre/eYFP	21% fat, 0.2% chol*	18
Kim	32441123	GSE150768	2 SMC tdT^{+}	AR, AA	ND	ApoE$^{-/-}$ Myh11-Cre^{ERT2}/tdT	0.25% chol **	16
Wirka	31359001	GSE131780	6 SMC tdT^{+}<br>5 Non-SMC tdT^{-}	AR, AA	ND	ApoE$^{-/-}$ Myh11-Cre^{ERT2}/tdT	21% fat, 0.15% chol ***	0, 8,16

SMC, smooth muscle cell; #, unsorted/all cell types; AA, Ascending aorta; BA, brachiocephalic artery; TA, Thoracic aorta; AR, aortic root; ND, not described; * TD.88137; ** Tecnilab-BMI 824171; *** Dyets 101511

Supplementary Table 2: Counts after quality control in CellRanger software

**Parameters**	***Ldlr$^{-/-}$*, adventitia, standard laboratory diet**	***Ldlr$^{-/-}$*, adventitia, 16 weeks HCD**	***Ldlr$^{-/-}$*, plaque, 16 weeks HCD**
Estimated # of cells	4,822	7,989	3,976
Mean reads per cell	63,948	47,390	34,531
Median genes per cell	2,359	1,640	2,151
Median UMI count per cell	6,794	3,545	5,513

Supplementary Table 3: Antibody information

Antibody (Product code, company)	IHC/flow	HIER	Blocking	Dilution	Clone name	Lot no.	Validation statement (company statement (i.e KD/KO validated, or some other text, or reference to papers showing specificity
tdTomato (#orb182397, Biorbyt)	IHC	ND	ND	1:2000	Polyclonal	AR2162	Antibody was validated based on information provided by the vendor: https://www.biorbyt.com/tdtomato-antibody-orb182397.html. Validated (Ex. Fig. 4. I.; PMID: 35728625), and in house Fig. 3. C. D.
Mouse CD31 (#ab182981, Abcam)	IHC	pH high *	5% NGS	1:5000	EPR17259	1075935-8	Antibody was validated based on information provided by the vendor: https://www.abcam.com/en-us/products/primary-antibodies/cd31-antibody-epr17259-ab182981. Abcam “Advanced Validation” certification on product sheet.
SM22α (#ab10135, Abcam)	IHC	pH Low **	20% NDS	1:400	Polyclonal	1015582-4	Antibody was validated based on information provided by the vendor: https://www.abcam.com/en-us/products/primary-antibodies/tagln-transgelin-antibody-ab10135
MYH11 (ab53219, Abcam)	IHC	pH Low **	10% NSS	1:100	Polyclonal	NA (discontinued)	Antibody was validated based on information provided by the vendor available via CiteAb: https://www.citeab.com/antibodies/774060-ab53219-anti-smooth-muscle-myosin-heavy-chain-11-anti KO validated (PMID: 37468521)
ACTA2 (#M0851, Dako)	IHC	pH high *	10% NDS	1:500	Monoclonal (1A4)	41648789	Antibody was validated based on information provided by the vendor: https://www.agilent.com/en/product/immunohistochemistry/antibodies-controls/primary-antibodies/actin-%28smooth-muscle%29-%28concentrate%29-76542
UGDH (#PA5-88347, Invitrogen, ThermoFisher)	IHC	pH Low **	10% NGS	1:400	Polyclonal	XI3692164A	Antibody was validated based on information provided by the vendor: https://www.thermofisher.com/antibody/product/UGDH-Antibody-Polyclonal/PA5-88347
CD68 (#ab125212, Abcam)	IHC	pH high *	ND	1:500	Polyclonal	GR3326063-1	Antibody was validated based on information provided by the vendor: https://www.abcam.com/en-us/products/primary-antibodies/cd68-antibody-ab125212#
PDGFRa (#BAF1062, Biotechne)	IHC	pH Low **	20% NDS	1:50	Polyclonal	HNR0322101	Antibody was validated based on information provided by the vendor: https://www.bio-techne.com/p/antibodies/mouse-pdgf-ralpha-biotinylated-antibody_baf1062#tab-technical_data
PCNA abcam	IHC	PH Low	ND	1:500	Monoclonal (EPR3821)	108843-33	Antibody was validated by staining murine intestine paraffin section and co-staining with DAPI. Showed specific signal in the intestinal crypts. Used in over 420 publications according to the vendor: https://www.abcam.com/en-us/products/primary-antibodies/pcna-antibody-epr3821-ab92552
Pi16 (Proteintech 12267-1-AP)	IHC	PH Low	2.5% NHS	1:500	Polyclonal	00129310 41236479	Antibody was validated by western blot (vendor), KD validated (vendor) and cellular location and comparable, specific signal in mouse testis as human testis on human proteome atlas images. Used in 2 publications according to the vendor: https://www.ptglab.com/products/PI16-Antibody-12267-1-

							AP.htm?srsltid=AfmBOopJQ7LXsYmRuu7Q0QF6oLHWv_Z6ql2Bfflxm48snRu5-qUtxLg0#publications
Human CD31 (Dako IR610)	IHC	PH Low	2.5% NHS	Ready to use	Monoclonal	41236479	Antibody was validated by staining arteries, capillaries and microvessels across organs. Use in routine diagnostics, and 4 publications according to the vendor: https://www.agilent.com/en/product/immunohistochemistry/antibodies-controls/primary-antibodies/cd31-endothelial-cell-(autostainer-link-48)-76341?srsltid=AfmBOopXujnTZ_oQTnTYf9KtlNl41ltGgxnpX-yTvSVRrE-Rclp8kO5w#references
Human PDGFRA Invitrogen MA5-41209	IHC					ZG4393634	Antibody was validated by western blot (vendor), KD validated (vendor) and on IHC cellular location and signal were comparable to human kidney on human proteome atlas images.
Cdh5-AF657 (BD biosciences 561567)	Flow in vitro			1:200	Monoclonal (55-7H1)	320189	Antibody was validated based on information provided by the vendor: https://www.citeab.com/antibodies/2409031-561567-bd-pharmingen-alexa-fluor-647-mouse-anti-hu and PMID: 33972531
CD45 APC-Fire750 (Biolegend 103154)	Flow adv			1:100	Monoclonal (30-F11)	B351504	Antibody was validated based on information provided by the vendor: https://www.citeab.com/antibodies/3388400-103154-apc-fire-750-anti-mouse-cd45-antibody and PMID: 37316667
CDH5 PerCP-eFLuor710 (eBioscience 46-1441-82)	Flow adv			1:100	Monoclonal (BV13)	2464394	Antibody was validated based on information provided by the vendor: https://www.thermofisher.com/antibody/product/CD144-VE-cadherin-Antibody-clone-eBioBV13-BV13-Monoclonal/46-1441-82 and PMID: 24762086
PDGFRa BV421 (BD Pharmingen 562774)	Flow adv			1:25	Monoclonal (APA5)	1277587	Antibody was validated based on information provided by the vendor: https://www.bdbiosciences.com/en-eu/products/reagents/flow-cytometry-reagents/research-reagents/single-color-antibodies-ruo/bv421-rat-anti-mouse-cd140a.562774 and PMID: 38123537
Transgelin exaFluor488 (Novus biologicals NBP2-47689AF488)	Flow adv intracellular			1:100	Monoclonal (SPM606)	D107146	Antibody was validated based on information provided by the vendor: https://www.novusbio.com/products/transgelin-tagln-sm22-alpha-antibody-spm606_nbp2-47689af488
CD16/32 (eBioscience/Thermofisher 14-0161-85)	Flow blood			1:100	Monoclonal (93)	2071500	Antibody was validated based on information provided by the vendor: https://www.thermofisher.com/order/genome-database/generatePdf?productName=CD16/CD32&assayType=ANTIBODY&productId=14-0161-85&detailed=true and PMID: 32023474
CD45 PerCP (Biolegend 103130)	Flow blood			1:100	Monoclonal (30-F11)	B236192	Antibody was validated based on information provided by the vendor: https://www.biolegend.com/fr-ch/products/percp-anti-mouse-cd45-antibody-4265?GroupID=BLG6833 and https://www.citeab.com/antibodies/518036-103130-percp-anti-mouse-cd45-antibody and PMID: 35483404

IHC, immunohistochemistry; flow, flow cytometry; adv, adventitia; HIER, Heat-induced epitope retrieval; ND, not done; *, DaKo EnVision FLEX Target Retrieval Solution, #GV800, **, EnVision FLEX Target Retrieval Solution, #K80050; NGS, normal goat serum (#X0907, DAKO); NDS, normal donkey serum (#AB7475, Abcam); NSS normal swine serum (S-4000-20, Vector), NHS, Normal Horse Serum (DK-2488, Vector). NA, Not available.

Supplementary Table 4: Sequences of mouse primers used for RT-qPCR

**Gene**	**Species**	**Forward primer 5'-3'**	**Reverse primer 5'-3'**
Pdgfra	*Mouse*	AGAGAGAATCGGCCCCAGTG	CCATAGCTCCTGAGACCCGC
Myh11	*Mouse*	TGGACACCATGTCAGGGAAA	ATGGACACAAGTGCTAAGCAGTCT
Acta2	*Mouse*	GACTACTGCCGAGCGTGAG	ATAGGTGGTTTCGTGGATGC
PECAM1	*Mouse*	AGCCAACAGCCATTACGGTTA	AGCCTTCCGTTCTCTTGGTG
Cd68	*Mouse*	CGG CTC CCT GTG TGT CTG AT	CGT AGG GCT GGC TGT GCT T
Gapdh	*Mouse*	CAACTCACTCAAGATTGTCAGCAA	TGGCAGTGATGGCATGGA
GAPDH	*Human*	AGCCACATCGCTCAGACAC	GCCCAATACGACCAAATCC
Morf4l1	*Human*	AATGGGTTCCGGAGAGCAG	CTGCATACTGCTCCTGATTGG
PECAM1	*Human*	GCAACACAGTCCAGATAGTCGT	GACCTCAAACTGGGCATCAT
CDH5	*Human*	AAGCCTCTGATTGGCACAGT	CTGGCCCTTGTCACTGGT
PDGFRa	*Human*	GACTAGTGCTTGGTCGGGTC	CAGGTTGGGACCGGCTTAAT
Tagln	*Human*	ATAGTGCAGTGTGGCCCTGA	AGCTTGCTCAGAATCACGCC
ACTA2	*Human*	CTGTTCCAGCCATCCTTCAT	TCATGATGCTGTTGTAGGTGGT
CD68	*Human*	AGCTACATGGCGGTGGAGT	GATGAGAGGCAGCAAG

Supplementary Table 5: Acta2 mRNA expression

	Acta2<br>(% of *Myh11* Lin$^+$)	Acta2<br>(% of *Myh11* Lin$^-$)
SMC	99 (n = 16309)	96 (n = 1006)
Fibromyocyte	93 (n = 3941)	53 (n = 2778)
Chondromyocyte	77 (n = 415)	44 (n = 330)
Fibroblast (total)	87 (n = 604)	33 (n = 9698)
Matrix-fibroblast	n.a.	39
Progenitor fibroblast	n.a.	14
Inflammatory fibroblast	n.a.	27
EC-like fibroblast	n.a.	40

n.a. not available, per analysis, only *Myh11* Lin$^-$ were included in subset analysis.

Supplementary Table 6: GO analysis of murine fibroblast subsets

(separate excel spreadsheet uploaded).

Supplementary Table 7: GO analysis of human fibroblast subsets

(separate excel spreadsheet uploaded).
